# Supplementary material for: Thermal acclimation of tropical coral reef fishes to global heat waves
Source: eLife. 2021 Jan 26;10:e59162. doi: 10.7554/eLife.59162 (PMC7837695; doi:10.7554/eLife.59162)
Supplement: Supplementary file 1. [file elife-59162-supp1.docx]

Supplemental information Johansen & Nadler et al.

Statistical model outputs

**Respirometry data (SMR, MMR, Aerobic Scope)**

***Model:*** Species*Treatment*Measurement + (Mass|Species:fish)

***Overall Results:***

|  | f | SumSq | MeanSq | Fvalue | upper.df | upper.p | lower.df | lower.p | expl.dev.(%) |
| --- | --- | --- | --- | --- | --- | --- | --- | --- | --- |
| Species | 1 | 0.0012 | 0.0012 | 0.0002 | 293 | 0.9884 | 109 | 0.9884 | 0.0000 |
| Treatment | 6 | 228.2445 | 38.0407 | 6.9462 | 293 | 0.0000 | 109 | 0.0000 | 2.1309 |
| Measurement | 2 | 6170.3311 | 3085.1655 | 563.3513 | 293 | 0.0000 | 109 | 0.0000 | 57.6061 |
| Species : Treatment | 5 | 324.8250 | 64.9650 | 11.8626 | 293 | 0.0000 | 109 | 0.0000 | 3.0326 |
| Species : Measurement | 2 | 145.8772 | 72.9386 | 13.3186 | 293 | 0.0000 | 109 | 0.0000 | 1.3619 |
| Treatment : Measurement | 12 | 151.1387 | 12.5949 | 2.2998 | 293 | 0.0082 | 109 | 0.0118 | 1.4110 |
| Species : Treatment : Measurement | 10 | 140.4836 | 14.0484 | 2.5652 | 293 | 0.0055 | 109 | 0.0080 | 1.3116 |

***Model derived mass adjusted least squares means of transformed data (Coxbox exponent 0.5)***

$lsmeans

Treatment Measure Species lsmean SE df lower.CL upper.CL

Control AS Apogonid 17.61523 0.7330610 140.96 16.166020 19.06445

HT wk 0 AS Apogonid 24.42085 0.8397196 133.45 22.759970 26.08173

HT wk 1 AS Apogonid 24.40764 1.0540611 157.09 22.325674 26.48960

HT wk 2 AS Apogonid 22.04524 1.1341683 143.72 19.803437 24.28705

HT wk 3 AS Apogonid 16.38318 1.1446986 131.62 14.118792 18.64757

HT wk 4 AS Apogonid 17.65963 1.4734711 180.07 14.752137 20.56712

HT wk 5 AS Apogonid NA NA NA NA NA

Control MMR Apogonid 22.27776 0.7330610 140.96 20.828543 23.72697

HT wk 0 MMR Apogonid 29.20357 0.8397196 133.45 27.542691 30.86445

HT wk 1 MMR Apogonid 28.10105 0.9571863 135.10 26.208044 29.99406

HT wk 2 MMR Apogonid 26.37011 1.0712200 129.99 24.250828 28.48939

HT wk 3 MMR Apogonid 18.91951 1.1446986 131.62 16.655123 21.18390

HT wk 4 MMR Apogonid 20.71632 1.2350548 137.41 18.274152 23.15849

HT wk 5 MMR Apogonid NA NA NA NA NA

Control SMR Apogonid 13.34251 0.7330610 140.96 11.893297 14.79173

HT wk 0 SMR Apogonid 15.38798 0.8397196 133.45 13.727103 17.04887

HT wk 1 SMR Apogonid 13.48392 1.0540611 157.09 11.401964 15.56589

HT wk 2 SMR Apogonid 13.27828 1.1341683 143.72 11.036474 15.52008

HT wk 3 SMR Apogonid 9.14411 1.1446986 131.62 6.879722 11.40850

HT wk 4 SMR Apogonid 11.64391 1.4734711 180.07 8.736416 14.55140

HT wk 5 SMR Apogonid NA NA NA NA NA

Control AS Caesionid 17.75422 1.0345179 112.33 15.704518 19.80392

HT wk 0 AS Caesionid 18.87599 0.9947502 107.76 16.904178 20.84781

HT wk 1 AS Caesionid 19.05100 1.0574200 99.14 16.952885 21.14911

HT wk 2 AS Caesionid 20.49476 1.0292183 106.94 18.454438 22.53507

HT wk 3 AS Caesionid 21.21567 0.9937185 96.34 19.243247 23.18810

HT wk 4 AS Caesionid 19.89183 1.1224070 115.69 17.668696 22.11496

HT wk 5 AS Caesionid 18.33344 1.0532182 87.69 16.240282 20.42659

Control MMR Caesionid 22.67291 1.0345179 112.33 20.623208 24.72261

HT wk 0 MMR Caesionid 25.75915 0.9947502 107.76 23.787335 27.73097

HT wk 1 MMR Caesionid 24.78625 1.0574200 99.14 22.688134 26.88436

HT wk 2 MMR Caesionid 25.26265 1.0292183 106.94 23.222333 27.30297

HT wk 3 MMR Caesionid 26.27098 0.9937185 96.34 24.298551 28.24340

HT wk 4 MMR Caesionid 24.97073 1.1224070 115.69 22.747603 27.19387

HT wk 5 MMR Caesionid 23.84058 1.0532182 87.69 21.747425 25.93373

Control SMR Caesionid 13.98081 1.0345179 112.33 11.931110 16.03051

HT wk 0 SMR Caesionid 17.32649 0.9947502 107.76 15.354674 19.29831

HT wk 1 SMR Caesionid 15.54736 1.0574200 99.14 13.449248 17.64548

HT wk 2 SMR Caesionid 14.62367 1.0292183 106.94 12.583347 16.66398

HT wk 3 SMR Caesionid 15.34148 0.9937185 96.34 13.369049 17.31390

HT wk 4 SMR Caesionid 14.68207 1.1224070 115.69 12.458934 16.90520

HT wk 5 SMR Caesionid 14.98566 1.0532182 87.69 12.892511 17.07882

***Planned contrast comparisons with FDR corrections for Type II error***

*(Pcutoff = 0.0195 for apogonids and 0.0212 for fusileras)*

$contrasts

contrast estimate SE df t.ratio p.value

Control,AS,Apogonid - HT wk 0,AS,Apogonid -6.80561744 1.1146782 136.75 -6.105 <.0001

Control,AS,Apogonid - HT wk 1,AS,Apogonid -6.79240024 1.2839094 151.78 -5.290 <.0001

Control,AS,Apogonid - HT wk 2,AS,Apogonid -4.43000748 1.3504503 143.12 -3.280 0.0013

Control,AS,Apogonid - HT wk 3,AS,Apogonid 1.23205454 1.3593062 134.39 0.906 0.3664

Control,AS,Apogonid - HT wk 4,AS,Apogonid -0.04439271 1.6457508 171.73 -0.027 0.9785

Control,AS,Apogonid - HT wk 5,AS,Apogonid NA NA NA NA NA

HT wk 0,AS,Apogonid - HT wk 1,AS,Apogonid 0.01321719 1.3476550 147.46 0.010 0.9922

HT wk 0,AS,Apogonid - HT wk 2,AS,Apogonid 2.37560996 1.4111934 140.02 1.683 0.0945

HT wk 0,AS,Apogonid - HT wk 3,AS,Apogonid 8.03767197 1.4196704 132.26 5.662 <.0001

HT wk 0,AS,Apogonid - HT wk 4,AS,Apogonid 6.76122472 1.6959499 167.54 3.987 0.0001

HT wk 0,AS,Apogonid - HT wk 5,AS,Apogonid NA NA NA NA NA

HT wk 1,AS,Apogonid - HT wk 2,AS,Apogonid 2.36239276 1.5483483 149.79 1.526 0.1292

HT wk 1,AS,Apogonid - HT wk 3,AS,Apogonid 8.02445478 1.5560783 142.75 5.157 <.0001

HT wk 1,AS,Apogonid - HT wk 4,AS,Apogonid 6.74800753 1.8116738 172.06 3.725 0.0003

HT wk 1,AS,Apogonid - HT wk 5,AS,Apogonid NA NA NA NA NA

HT wk 2,AS,Apogonid - HT wk 3,AS,Apogonid 5.66206201 1.6114195 137.49 3.514 0.0006

HT wk 2,AS,Apogonid - HT wk 4,AS,Apogonid 4.38561477 1.8594232 165.84 2.359 0.0195

HT wk 2,AS,Apogonid - HT wk 5,AS,Apogonid NA NA NA NA NA

HT wk 3,AS,Apogonid - HT wk 4,AS,Apogonid -1.27644725 1.8658650 160.29 -0.684 0.4949

HT wk 3,AS,Apogonid - HT wk 5,AS,Apogonid NA NA NA NA NA

HT wk 4,AS,Apogonid - HT wk 5,AS,Apogonid NA NA NA NA NA

Control,MMR,Apogonid - HT wk 0,MMR,Apogonid -6.92581468 1.1146782 136.75 -6.213 <.0001

Control,MMR,Apogonid - HT wk 1,MMR,Apogonid -5.82329336 1.2056467 137.30 -4.830 <.0001

Control,MMR,Apogonid - HT wk 2,MMR,Apogonid -4.09235113 1.2980334 133.57 -3.153 0.0020

Control,MMR,Apogonid - HT wk 3,MMR,Apogonid 3.35824726 1.3593062 134.39 2.471 0.0147

Control,MMR,Apogonid - HT wk 4,MMR,Apogonid 1.56143541 1.4362238 138.35 1.087 0.2788

Control,MMR,Apogonid - HT wk 5,MMR,Apogonid NA NA NA NA NA

HT wk 0,MMR,Apogonid - HT wk 1,MMR,Apogonid 1.10252132 1.2733164 134.38 0.866 0.3881

HT wk 0,MMR,Apogonid - HT wk 2,MMR,Apogonid 2.83346356 1.3611177 131.31 2.082 0.0393

HT wk 0,MMR,Apogonid - HT wk 3,MMR,Apogonid 10.28406194 1.4196704 132.26 7.244 <.0001

HT wk 0,MMR,Apogonid - HT wk 4,MMR,Apogonid 8.48725009 1.4934823 136.17 5.683 <.0001

HT wk 0,MMR,Apogonid - HT wk 5,MMR,Apogonid NA NA NA NA NA

HT wk 1,MMR,Apogonid - HT wk 2,MMR,Apogonid 1.73094223 1.4365646 132.27 1.205 0.2304

HT wk 1,MMR,Apogonid - HT wk 3,MMR,Apogonid 9.18154062 1.4921597 133.05 6.153 <.0001

HT wk 1,MMR,Apogonid - HT wk 4,MMR,Apogonid 7.38472877 1.5625512 136.55 4.726 <.0001

HT wk 1,MMR,Apogonid - HT wk 5,MMR,Apogonid NA NA NA NA NA

HT wk 2,MMR,Apogonid - HT wk 3,MMR,Apogonid 7.45059838 1.5677523 130.86 4.752 <.0001

HT wk 2,MMR,Apogonid - HT wk 4,MMR,Apogonid 5.65378654 1.6348923 134.26 3.458 0.0007

HT wk 2,MMR,Apogonid - HT wk 5,MMR,Apogonid NA NA NA NA NA

HT wk 3,MMR,Apogonid - HT wk 4,MMR,Apogonid -1.79681185 1.6839523 134.76 -1.067 0.2879

HT wk 3,MMR,Apogonid - HT wk 5,MMR,Apogonid NA NA NA NA NA

HT wk 4,MMR,Apogonid - HT wk 5,MMR,Apogonid NA NA NA NA NA

Control,SMR,Apogonid - HT wk 0,SMR,Apogonid -2.04547279 1.1146782 136.75 -1.835 0.0687

Control,SMR,Apogonid - HT wk 1,SMR,Apogonid -0.14141270 1.2839094 151.78 -0.110 0.9124

Control,SMR,Apogonid - HT wk 2,SMR,Apogonid 0.06423295 1.3504503 143.12 0.048 0.9621

Control,SMR,Apogonid - HT wk 3,SMR,Apogonid 4.19840152 1.3593062 134.39 3.089 0.0024

Control,SMR,Apogonid - HT wk 4,SMR,Apogonid 1.69860495 1.6457508 171.73 1.032 0.3035

Control,SMR,Apogonid - HT wk 5,SMR,Apogonid NA NA NA NA NA

HT wk 0,SMR,Apogonid - HT wk 1,SMR,Apogonid 1.90406009 1.3476550 147.46 1.413 0.1598

HT wk 0,SMR,Apogonid - HT wk 2,SMR,Apogonid 2.10970574 1.4111934 140.02 1.495 0.1372

HT wk 0,SMR,Apogonid - HT wk 3,SMR,Apogonid 6.24387431 1.4196704 132.26 4.398 <.0001

HT wk 0,SMR,Apogonid - HT wk 4,SMR,Apogonid 3.74407774 1.6959499 167.54 2.208 0.0286

HT wk 0,SMR,Apogonid - HT wk 5,SMR,Apogonid NA NA NA NA NA

HT wk 1,SMR,Apogonid - HT wk 2,SMR,Apogonid 0.20564565 1.5483483 149.79 0.133 0.8945

HT wk 1,SMR,Apogonid - HT wk 3,SMR,Apogonid 4.33981422 1.5560783 142.75 2.789 0.0060

HT wk 1,SMR,Apogonid - HT wk 4,SMR,Apogonid 1.84001765 1.8116738 172.06 1.016 0.3112

HT wk 1,SMR,Apogonid - HT wk 5,SMR,Apogonid NA NA NA NA NA

HT wk 2,SMR,Apogonid - HT wk 3,SMR,Apogonid 4.13416857 1.6114195 137.49 2.566 0.0114

HT wk 2,SMR,Apogonid - HT wk 4,SMR,Apogonid 1.63437200 1.8594232 165.84 0.879 0.3807

HT wk 2,SMR,Apogonid - HT wk 5,SMR,Apogonid NA NA NA NA NA

HT wk 3,SMR,Apogonid - HT wk 4,SMR,Apogonid -2.49979657 1.8658650 160.29 -1.340 0.1822

HT wk 3,SMR,Apogonid - HT wk 5,SMR,Apogonid NA NA NA NA NA

HT wk 4,SMR,Apogonid - HT wk 5,SMR,Apogonid NA NA NA NA NA

Control,AS,Caesionid - HT wk 0,AS,Caesionid -1.12177672 1.4351848 137.58 -0.782 0.4358

Control,AS,Caesionid - HT wk 1,AS,Caesionid -1.29678216 1.4793121 120.53 -0.877 0.3824

Control,AS,Caesionid - HT wk 2,AS,Caesionid -2.74053829 1.4592867 153.52 -1.878 0.0623

Control,AS,Caesionid - HT wk 3,AS,Caesionid -3.46145624 1.4344699 156.86 -2.413 0.0170

Control,AS,Caesionid - HT wk 4,AS,Caesionid -2.13761060 1.5264419 155.29 -1.400 0.1634

Control,AS,Caesionid - HT wk 5,AS,Caesionid -0.57921772 1.4763116 170.98 -0.392 0.6953

HT wk 0,AS,Caesionid - HT wk 1,AS,Caesionid -0.17500544 1.4517799 104.63 -0.121 0.9043

HT wk 0,AS,Caesionid - HT wk 2,AS,Caesionid -1.61876157 1.4313693 108.97 -1.131 0.2606

HT wk 0,AS,Caesionid - HT wk 3,AS,Caesionid -2.33967952 1.1712719 212.02 -1.998 0.0470

HT wk 0,AS,Caesionid - HT wk 4,AS,Caesionid -1.01583388 1.4997751 113.13 -0.677 0.4996

HT wk 0,AS,Caesionid - HT wk 5,AS,Caesionid 0.54255900 1.4487224 105.11 0.375 0.7088

HT wk 1,AS,Caesionid - HT wk 2,AS,Caesionid -1.44375613 1.4756108 109.11 -0.978 0.3300

HT wk 1,AS,Caesionid - HT wk 3,AS,Caesionid -2.16467408 1.4510732 107.14 -1.492 0.1387

HT wk 1,AS,Caesionid - HT wk 4,AS,Caesionid -0.84082844 1.3129411 213.96 -0.640 0.5226

HT wk 1,AS,Caesionid - HT wk 5,AS,Caesionid 0.71756444 1.4924495 110.88 0.481 0.6316

HT wk 2,AS,Caesionid - HT wk 3,AS,Caesionid -0.72091794 1.4306525 101.77 -0.504 0.6154

HT wk 2,AS,Caesionid - HT wk 4,AS,Caesionid 0.60292769 1.5228552 111.99 0.396 0.6929

HT wk 2,AS,Caesionid - HT wk 5,AS,Caesionid 2.16132058 1.1733406 211.99 1.842 0.0669

HT wk 3,AS,Caesionid - HT wk 4,AS,Caesionid 1.32384564 1.4990911 107.53 0.883 0.3792

HT wk 3,AS,Caesionid - HT wk 5,AS,Caesionid 2.88223852 1.4480142 92.94 1.990 0.0495

HT wk 4,AS,Caesionid - HT wk 5,AS,Caesionid 1.55839288 1.5391771 106.06 1.012 0.3136

Control,MMR,Caesionid - HT wk 0,MMR,Caesionid -3.08624291 1.4351848 137.58 -2.150 0.0333

Control,MMR,Caesionid - HT wk 1,MMR,Caesionid -2.11334140 1.4793121 120.53 -1.429 0.1557

Control,MMR,Caesionid - HT wk 2,MMR,Caesionid -2.58974366 1.4592867 153.52 -1.775 0.0779

Control,MMR,Caesionid - HT wk 3,MMR,Caesionid -3.59806965 1.4344699 156.86 -2.508 0.0131

Control,MMR,Caesionid - HT wk 4,MMR,Caesionid -2.29782701 1.5264419 155.29 -1.505 0.1343

Control,MMR,Caesionid - HT wk 5,MMR,Caesionid -1.16767052 1.4763116 170.98 -0.791 0.4301

HT wk 1,MMR,Caesionid - HT wk 2,MMR,Caesionid -0.47640226 1.4756108 109.11 -0.323 0.7474

HT wk 1,MMR,Caesionid - HT wk 3,MMR,Caesionid -1.48472825 1.4510732 107.14 -1.023 0.3085

HT wk 1,MMR,Caesionid - HT wk 4,MMR,Caesionid -0.18448561 1.3129411 213.96 -0.141 0.8884

HT wk 1,MMR,Caesionid - HT wk 5,MMR,Caesionid 0.94567088 1.4924495 110.88 0.634 0.5276

HT wk 2,MMR,Caesionid - HT wk 3,MMR,Caesionid -1.00832598 1.4306525 101.77 -0.705 0.4825

HT wk 2,MMR,Caesionid - HT wk 4,MMR,Caesionid 0.29191666 1.5228552 111.99 0.192 0.8483

HT wk 2,MMR,Caesionid - HT wk 5,MMR,Caesionid 1.42207315 1.1733406 211.99 1.212 0.2269

HT wk 3,MMR,Caesionid - HT wk 4,MMR,Caesionid 1.30024264 1.4990911 107.53 0.867 0.3877

HT wk 3,MMR,Caesionid - HT wk 5,MMR,Caesionid 2.43039913 1.4480142 92.94 1.678 0.0966

HT wk 4,MMR,Caesionid - HT wk 5,MMR,Caesionid 1.13015649 1.5391771 106.06 0.734 0.4644

Control,SMR,Caesionid - HT wk 0,SMR,Caesionid -3.34568013 1.4351848 137.58 -2.331 0.0212

Control,SMR,Caesionid - HT wk 1,SMR,Caesionid -1.56655276 1.4793121 120.53 -1.059 0.2917

Control,SMR,Caesionid - HT wk 2,SMR,Caesionid -0.64285564 1.4592867 153.52 -0.441 0.6602

Control,SMR,Caesionid - HT wk 3,SMR,Caesionid -1.36066601 1.4344699 156.86 -0.949 0.3443

Control,SMR,Caesionid - HT wk 4,SMR,Caesionid -0.70125638 1.5264419 155.29 -0.459 0.6466

Control,SMR,Caesionid - HT wk 5,SMR,Caesionid -1.00485416 1.4763116 170.98 -0.681 0.4970

HT wk 0,SMR,Caesionid - HT wk 1,SMR,Caesionid 1.77912737 1.4517799 104.63 1.225 0.2231

HT wk 0,SMR,Caesionid - HT wk 2,SMR,Caesionid 2.70282449 1.4313693 108.97 1.888 0.0616

HT wk 0,SMR,Caesionid - HT wk 3,SMR,Caesionid 1.98501411 1.1712719 212.02 1.695 0.0916

HT wk 0,SMR,Caesionid - HT wk 4,SMR,Caesionid 2.64442375 1.4997751 113.13 1.763 0.0806

HT wk 0,SMR,Caesionid - HT wk 5,SMR,Caesionid 2.34082597 1.4487224 105.11 1.616 0.1091

HT wk 1,SMR,Caesionid - HT wk 2,SMR,Caesionid 0.92369712 1.4756108 109.11 0.626 0.5326

HT wk 1,SMR,Caesionid - HT wk 3,SMR,Caesionid 0.20588675 1.4510732 107.14 0.142 0.8874

HT wk 1,SMR,Caesionid - HT wk 4,SMR,Caesionid 0.86529638 1.3129411 213.96 0.659 0.5106

HT wk 1,SMR,Caesionid - HT wk 5,SMR,Caesionid 0.56169860 1.4924495 110.88 0.376 0.7074

HT wk 2,SMR,Caesionid - HT wk 3,SMR,Caesionid -0.71781038 1.4306525 101.77 -0.502 0.6169

HT wk 2,SMR,Caesionid - HT wk 4,SMR,Caesionid -0.05840074 1.5228552 111.99 -0.038 0.9695

HT wk 2,SMR,Caesionid - HT wk 5,SMR,Caesionid -0.36199852 1.1733406 211.99 -0.309 0.7580

HT wk 3,SMR,Caesionid - HT wk 4,SMR,Caesionid 0.65940963 1.4990911 107.53 0.440 0.6609

HT wk 3,SMR,Caesionid - HT wk 5,SMR,Caesionid 0.35581186 1.4480142 92.94 0.246 0.8064

HT wk 4,SMR,Caesionid - HT wk 5,SMR,Caesionid -0.30359778 1.5391771 106.06 -0.197 0.8440

**Fusilera Blood Analyses**

***Model:*** Treatment*Tissue + (BM|Treatment:No)

***Overall Results:***

|  | f | SumSq | MeanSq | Fvalue | upper.df | upper.p | lower.df | lower.p | expl.dev.(%) |
| --- | --- | --- | --- | --- | --- | --- | --- | --- | --- |
| Treatment | 6 | 3.9376 | 0.6563 | 2.2159 | 549 | 0.0402 | 437 | 0.0406 | 0.1174 |
| Tissue | 11 | 3156.47 | 286.9520 | 968.9133 | 549 | 0.0000 | 437 | 0.0000 | 94.1089 |
| Treatment : Tissue | 66 | 27.4885 | 0.4165 | 1.4063 | 549 | 0.0238 | 437 | 0.0258 | 0.8196 |

***Model mass adjusted least squares means (LN transformation)***

$lsmeans

Treatment Tissue lsmean SE df lower.CL upper.CL

Control Blood [Hb] 2.01508024 0.2080348 543.52 1.60642955 2.42373094

Ht wk 0 Blood [Hb] 2.16787654 0.1937232 541.81 1.78733590 2.54841717

Ht wk 1 Blood [Hb] 2.22566448 0.2071832 542.91 1.81868551 2.63264344

Ht wk 2 Blood [Hb] 2.18765516 0.1944931 546.13 1.80560899 2.56970133

Ht wk 3 Blood [Hb] 2.21607704 0.1950741 539.81 1.83287969 2.59927439

Ht wk 4 Blood [Hb] 2.22088470 0.1946630 544.97 1.83850295 2.60326646

Ht wk 5 Blood [Hb] 2.09169377 0.2251623 480.08 1.64926841 2.53411912

Control Body condition 1.15408433 0.1947918 548.52 0.77145510 1.53671356

Ht wk 0 Body condition 0.95679736 0.1937232 541.81 0.57625673 1.33733799

Ht wk 1 Body condition 1.08346607 0.1936327 542.67 0.70310454 1.46382760

Ht wk 2 Body condition 0.89301432 0.1944931 546.13 0.51096815 1.27506049

Ht wk 3 Body condition 0.91144122 0.1950741 539.81 0.52824387 1.29463857

Ht wk 4 Body condition 0.94371262 0.1946630 544.97 0.56133086 1.32609437

Ht wk 5 Body condition 0.93531860 0.1942386 491.22 0.55367770 1.31695951

Control Gills CS 4.65936137 0.1947918 548.52 4.27673214 5.04199060

Ht wk 0 Gills CS 4.52415128 0.1937232 541.81 4.14361065 4.90469191

Ht wk 1 Gills CS 4.72889401 0.1936327 542.67 4.34853248 5.10925553

Ht wk 2 Gills CS 4.41983004 0.1944931 546.13 4.03778387 4.80187621

Ht wk 3 Gills CS 4.19845835 0.2089413 538.71 3.78801877 4.60889794

Ht wk 4 Gills CS 3.63963708 0.1946630 544.97 3.25725533 4.02201883

Ht wk 5 Gills CS 4.57011297 0.1942386 491.22 4.18847207 4.95175387

Control Gills LDH 5.61671900 0.1947918 548.52 5.23408977 5.99934823

Ht wk 0 Gills LDH 5.29931405 0.2227367 527.54 4.86175430 5.73687380

Ht wk 1 Gills LDH 5.28128183 0.2072233 543.58 4.87422534 5.68833831

Ht wk 2 Gills LDH 5.85280773 0.2066748 534.41 5.44681304 6.25880242

Ht wk 3 Gills LDH 5.76078482 0.2075254 537.14 5.35312387 6.16844578

Ht wk 4 Gills LDH 5.24376499 0.2249073 546.36 4.80197602 5.68555396

Ht wk 5 Gills LDH 5.46533074 0.1942386 491.22 5.08368984 5.84697164

Control Glucose 0.65862783 0.2080348 543.52 0.24997714 1.06727853

Ht wk 0 Glucose 0.75504766 0.2740575 530.72 0.21667706 1.29341825

Ht wk 1 Glucose 1.53746601 0.2071832 542.91 1.13048704 1.94444498

Ht wk 2 Glucose 1.28039711 0.1944931 546.13 0.89835094 1.66244327

Ht wk 3 Glucose 1.10231353 0.1950741 539.81 0.71911618 1.48551088

Ht wk 4 Glucose 1.27077111 0.1946630 544.97 0.88838936 1.65315286

Ht wk 5 Glucose 0.87108271 0.2080025 488.59 0.46239297 1.27977244

Control Hcrt (%) 3.53485356 0.2080348 543.52 3.12620286 3.94350426

Ht wk 0 Hcrt (%) 3.66967515 0.1937232 541.81 3.28913451 4.05021578

Ht wk 1 Hcrt (%) 3.66757918 0.2237823 537.11 3.22798330 4.10717506

Ht wk 2 Hcrt (%) 3.64052590 0.1944931 546.13 3.25847973 4.02257207

Ht wk 3 Hcrt (%) 3.61165574 0.1950741 539.81 3.22845839 3.99485309

Ht wk 4 Hcrt (%) 3.54803809 0.1946630 544.97 3.16565634 3.93041984

Ht wk 5 Hcrt (%) 3.33790607 0.2251623 480.08 2.89548072 3.78033143

Control Lactate -0.35529517 0.2080348 543.52 -0.76394586 0.05335553

Ht wk 0 Lactate -0.12990862 0.1937232 541.81 -0.51044925 0.25063201

Ht wk 1 Lactate -0.09421849 0.2071832 542.91 -0.50119745 0.31276048

Ht wk 2 Lactate -0.22130876 0.1944931 546.13 -0.60335493 0.16073741

Ht wk 3 Lactate -0.21396141 0.2088336 542.24 -0.62418335 0.19626052

Ht wk 4 Lactate -0.23171806 0.1946630 544.97 -0.61409982 0.15066369

Ht wk 5 Lactate -0.20652662 0.2080025 488.59 -0.61521636 0.20216311

Control MCHC 1.24874838 0.2080348 543.52 0.84009768 1.65739907

Ht wk 0 MCHC 1.25138200 0.1937232 541.81 0.87084136 1.63192263

Ht wk 1 MCHC 1.32516318 0.2237823 537.11 0.88556730 1.76475906

Ht wk 2 MCHC 1.32771949 0.1944931 546.13 0.94567332 1.70976566

Ht wk 3 MCHC 1.37174199 0.1950741 539.81 0.98854464 1.75493935

Ht wk 4 MCHC 1.42753373 0.1946630 544.97 1.04515198 1.80991548

Ht wk 5 MCHC 1.51938530 0.2251623 480.08 1.07695994 1.96181065

Control Red Muscle CS 5.34753021 0.1947918 548.52 4.96490098 5.73015944

Ht wk 0 Red Muscle CS 5.36701207 0.1937232 541.81 4.98647143 5.74755270

Ht wk 1 Red Muscle CS 5.51863842 0.1936327 542.67 5.13827689 5.89899995

Ht wk 2 Red Muscle CS 5.28692714 0.1944931 546.13 4.90488097 5.66897331

Ht wk 3 Red Muscle CS 5.21601347 0.1950741 539.81 4.83281612 5.59921082

Ht wk 4 Red Muscle CS 4.64451395 0.1946630 544.97 4.26213220 5.02689570

Ht wk 5 Red Muscle CS 5.16268644 0.1942386 491.22 4.78104553 5.54432734

Control Red Muscle LDH 0.31036114 0.1947918 548.52 -0.07226809 0.69299038

Ht wk 0 Red Muscle LDH -0.50164843 0.1937232 541.81 -0.88218906 -0.12110780

Ht wk 1 Red Muscle LDH 0.11467767 0.2070060 540.12 -0.29195784 0.52131319

Ht wk 2 Red Muscle LDH -0.20285198 0.2082993 547.51 -0.61201553 0.20631156

Ht wk 3 Red Muscle LDH 0.60393503 0.2085978 545.73 0.19418203 1.01368802

Ht wk 4 Red Muscle LDH 0.46604067 0.1946630 544.97 0.08365892 0.84842242

Ht wk 5 Red Muscle LDH 0.40906108 0.1942386 491.22 0.02742018 0.79070198

Control Spleen [Hb] 1.71355305 0.1947918 548.52 1.33092382 2.09618228

Ht wk 0 Spleen [Hb] 1.55103728 0.1937232 541.81 1.17049664 1.93157791

Ht wk 1 Spleen [Hb] 1.81515978 0.2072532 544.11 1.40804548 2.22227408

Ht wk 2 Spleen [Hb] 1.82717359 0.1944931 546.13 1.44512742 2.20921976

Ht wk 3 Spleen [Hb] 2.07018602 0.1950741 539.81 1.68698867 2.45338337

Ht wk 4 Spleen [Hb] 2.13329784 0.1946630 544.97 1.75091609 2.51567959

Ht wk 5 Spleen [Hb] 2.31797289 0.2078753 504.19 1.90956434 2.72638143

Control SSI -2.23201892 0.1947918 548.52 -2.61464815 -1.84938969

Ht wk 0 SSI -2.18112900 0.1937232 541.81 -2.56166963 -1.80058836

Ht wk 1 SSI -2.28909804 0.1936327 542.67 -2.66945957 -1.90873651

Ht wk 2 SSI -2.23793067 0.2082008 546.71 -2.64690218 -1.82895915

Ht wk 3 SSI -2.34320432 0.1950741 539.81 -2.72640167 -1.96000697

Ht wk 4 SSI -2.18528233 0.1946630 544.97 -2.56766408 -1.80290058

Ht wk 5 SSI -2.07994890 0.1942386 491.22 -2.46158981 -1.69830800

***Planned contrast comparisons using FDR corrections for Type II error (Pcutoff = 0.0294)***

$contrasts

contrast estimate SE df t.ratio p.value

Control,Blood [Hb] - Ht wk 0,Blood [Hb] -0.1527962929 0.2842660 542.96 -0.538 0.5911

Control,Blood [Hb] - Ht wk 1,Blood [Hb] -0.2105842306 0.2936041 544.51 -0.717 0.4735

Control,Blood [Hb] - Ht wk 2,Blood [Hb] -0.1725749187 0.2847912 546.55 -0.606 0.5448

Control,Blood [Hb] - Ht wk 3,Blood [Hb] -0.2009967956 0.2851883 542.48 -0.705 0.4812

Control,Blood [Hb] - Ht wk 4,Blood [Hb] -0.2058044592 0.2849073 544.27 -0.722 0.4704

Control,Blood [Hb] - Ht wk 5,Blood [Hb] -0.0766135230 0.3065559 535.28 -0.250 0.8027

Ht wk 0,Blood [Hb] - Ht wk 1,Blood [Hb] -0.0577879377 0.2836434 543.02 -0.204 0.8386

Ht wk 0,Blood [Hb] - Ht wk 2,Blood [Hb] -0.0197786258 0.2745109 545.44 -0.072 0.9426

Ht wk 0,Blood [Hb] - Ht wk 3,Blood [Hb] -0.0482005026 0.2749229 542.52 -0.175 0.8609

Ht wk 0,Blood [Hb] - Ht wk 4,Blood [Hb] -0.0530081662 0.2746314 543.55 -0.193 0.8470

Ht wk 0,Blood [Hb] - Ht wk 5,Blood [Hb] 0.0761827700 0.2970299 532.41 0.256 0.7977

Ht wk 1,Blood [Hb] - Ht wk 2,Blood [Hb] 0.0380093119 0.2841698 544.82 0.134 0.8936

Ht wk 1,Blood [Hb] - Ht wk 3,Blood [Hb] 0.0095874351 0.2845677 545.23 0.034 0.9731

Ht wk 1,Blood [Hb] - Ht wk 4,Blood [Hb] 0.0047797715 0.2842861 544.65 0.017 0.9866

Ht wk 1,Blood [Hb] - Ht wk 5,Blood [Hb] 0.1339707077 0.3059787 529.92 0.438 0.6617

Ht wk 2,Blood [Hb] - Ht wk 3,Blood [Hb] -0.0284218768 0.2754659 546.98 -0.103 0.9179

Ht wk 2,Blood [Hb] - Ht wk 4,Blood [Hb] -0.0332295404 0.2751750 546.71 -0.121 0.9039

Ht wk 2,Blood [Hb] - Ht wk 5,Blood [Hb] 0.0959613958 0.2975326 523.40 0.323 0.7472

Ht wk 3,Blood [Hb] - Ht wk 4,Blood [Hb] -0.0048076636 0.2755859 543.51 -0.017 0.9861

Ht wk 3,Blood [Hb] - Ht wk 5,Blood [Hb] 0.1243832726 0.2979127 533.51 0.418 0.6765

Ht wk 4,Blood [Hb] - Ht wk 5,Blood [Hb] 0.1291909362 0.2976437 530.99 0.434 0.6644

Control,Body condition - Ht wk 0,Body condition 0.1972869750 0.2747227 547.05 0.718 0.4730

Control,Body condition - Ht wk 1,Body condition 0.0706182637 0.2746589 546.87 0.257 0.7972

Control,Body condition - Ht wk 2,Body condition 0.2610700120 0.2752661 547.77 0.948 0.3433

Control,Body condition - Ht wk 3,Body condition 0.2426431114 0.2756769 546.89 0.880 0.3792

Control,Body condition - Ht wk 4,Body condition 0.2103717196 0.2753862 547.86 0.764 0.4452

Control,Body condition - Ht wk 5,Body condition 0.2187657305 0.2750863 531.97 0.795 0.4268

Ht wk 0,Body condition - Ht wk 1,Body condition -0.1266687113 0.2739021 543.52 -0.462 0.6439

Ht wk 0,Body condition - Ht wk 2,Body condition 0.0637830371 0.2745109 545.44 0.232 0.8164

Ht wk 0,Body condition - Ht wk 3,Body condition 0.0453561364 0.2749229 542.52 0.165 0.8690

Ht wk 0,Body condition - Ht wk 4,Body condition 0.0130847446 0.2746314 543.55 0.048 0.9620

Ht wk 0,Body condition - Ht wk 5,Body condition 0.0214787555 0.2743307 539.63 0.078 0.9376

Ht wk 1,Body condition - Ht wk 2,Body condition 0.1904517483 0.2744471 544.64 0.694 0.4880

Ht wk 1,Body condition - Ht wk 3,Body condition 0.1720248476 0.2748591 546.09 0.626 0.5317

Ht wk 1,Body condition - Ht wk 4,Body condition 0.1397534559 0.2745675 545.17 0.509 0.6110

Ht wk 1,Body condition - Ht wk 5,Body condition 0.1481474668 0.2742668 531.87 0.540 0.5893

Ht wk 2,Body condition - Ht wk 3,Body condition -0.0184269007 0.2754659 546.98 -0.067 0.9467

Ht wk 2,Body condition - Ht wk 4,Body condition -0.0506982924 0.2751750 546.71 -0.184 0.8539

Ht wk 2,Body condition - Ht wk 5,Body condition -0.0423042815 0.2748749 531.32 -0.154 0.8777

Ht wk 3,Body condition - Ht wk 4,Body condition -0.0322713917 0.2755859 543.51 -0.117 0.9068

Ht wk 3,Body condition - Ht wk 5,Body condition -0.0238773808 0.2752862 541.78 -0.087 0.9309

Ht wk 4,Body condition - Ht wk 5,Body condition 0.0083940109 0.2749951 538.86 0.031 0.9757

Control,Gills CS - Ht wk 0,Gills CS 0.1352100867 0.2747227 547.05 0.492 0.6228

Control,Gills CS - Ht wk 1,Gills CS -0.0695326397 0.2746589 546.87 -0.253 0.8002

Control,Gills CS - Ht wk 2,Gills CS 0.2395313214 0.2752661 547.77 0.870 0.3846

Control,Gills CS - Ht wk 3,Gills CS 0.4609030147 0.2856577 546.38 1.613 0.1072

Control,Gills CS - Ht wk 4,Gills CS 1.0197242858 0.2753862 547.86 3.703 0.0002

Control,Gills CS - Ht wk 5,Gills CS 0.0892483969 0.2750863 531.97 0.324 0.7457

Ht wk 0,Gills CS - Ht wk 1,Gills CS -0.2047427264 0.2739021 543.52 -0.748 0.4551

Ht wk 0,Gills CS - Ht wk 2,Gills CS 0.1043212347 0.2745109 545.44 0.380 0.7041

Ht wk 0,Gills CS - Ht wk 3,Gills CS 0.3256929280 0.2849301 543.30 1.143 0.2535

Ht wk 0,Gills CS - Ht wk 4,Gills CS 0.8845141990 0.2746314 543.55 3.221 0.0014

Ht wk 0,Gills CS - Ht wk 5,Gills CS -0.0459616898 0.2743307 539.63 -0.168 0.8670

Ht wk 1,Gills CS - Ht wk 2,Gills CS 0.3090639611 0.2744471 544.64 1.126 0.2606

Ht wk 1,Gills CS - Ht wk 3,Gills CS 0.5304356544 0.2848686 546.96 1.862 0.0631

Ht wk 1,Gills CS - Ht wk 4,Gills CS 1.0892569254 0.2745675 545.17 3.967 0.0001

Ht wk 1,Gills CS - Ht wk 5,Gills CS 0.1587810366 0.2742668 531.87 0.579 0.5629

Ht wk 2,Gills CS - Ht wk 3,Gills CS 0.2213716933 0.2854541 547.23 0.776 0.4384

Ht wk 2,Gills CS - Ht wk 4,Gills CS 0.7801929643 0.2751750 546.71 2.835 0.0047

Ht wk 2,Gills CS - Ht wk 5,Gills CS -0.1502829245 0.2748749 531.32 -0.547 0.5848

Ht wk 3,Gills CS - Ht wk 4,Gills CS 0.5588212710 0.2855699 543.78 1.957 0.0509

Ht wk 3,Gills CS - Ht wk 5,Gills CS -0.3716546178 0.2852807 541.88 -1.303 0.1932

Ht wk 4,Gills CS - Ht wk 5,Gills CS -0.9304758889 0.2749951 538.86 -3.384 0.0008

Control,Gills LDH - Ht wk 0,Gills LDH 0.3174049505 0.2958978 541.23 1.073 0.2839

Control,Gills LDH - Ht wk 1,Gills LDH 0.3354371748 0.2844035 547.05 1.179 0.2387

Control,Gills LDH - Ht wk 2,Gills LDH -0.2360887268 0.2840041 544.13 -0.831 0.4062

Control,Gills LDH - Ht wk 3,Gills LDH -0.1440658236 0.2846237 545.66 -0.506 0.6129

Control,Gills LDH - Ht wk 4,Gills LDH 0.3729540067 0.2975352 547.67 1.253 0.2106

Control,Gills LDH - Ht wk 5,Gills LDH 0.1513882614 0.2750863 531.97 0.550 0.5823

Ht wk 0,Gills LDH - Ht wk 1,Gills LDH 0.0180322243 0.3042254 536.22 0.059 0.9528

Ht wk 0,Gills LDH - Ht wk 2,Gills LDH -0.5534936773 0.3038521 530.95 -1.822 0.0691

Ht wk 0,Gills LDH - Ht wk 3,Gills LDH -0.4614707741 0.3044313 536.16 -1.516 0.1301

Ht wk 0,Gills LDH - Ht wk 4,Gills LDH 0.0555490562 0.3165359 540.74 0.175 0.8608

Ht wk 0,Gills LDH - Ht wk 5,Gills LDH -0.1660166891 0.2955338 532.95 -0.562 0.5745

Ht wk 1,Gills LDH - Ht wk 2,Gills LDH -0.5715259016 0.2926704 539.64 -1.953 0.0514

Ht wk 1,Gills LDH - Ht wk 3,Gills LDH -0.4795029984 0.2932717 542.36 -1.635 0.1026

Ht wk 1,Gills LDH - Ht wk 4,Gills LDH 0.0375168319 0.3058182 546.11 0.123 0.9024

Ht wk 1,Gills LDH - Ht wk 5,Gills LDH -0.1840489134 0.2840248 535.62 -0.648 0.5173

Ht wk 2,Gills LDH - Ht wk 3,Gills LDH 0.0920229032 0.2928844 539.86 0.314 0.7535

Ht wk 2,Gills LDH - Ht wk 4,Gills LDH 0.6090427336 0.3054469 542.99 1.994 0.0467

Ht wk 2,Gills LDH - Ht wk 5,Gills LDH 0.3874769882 0.2836249 531.15 1.366 0.1725

Ht wk 3,Gills LDH - Ht wk 4,Gills LDH 0.5170198303 0.3060231 546.80 1.689 0.0917

Ht wk 3,Gills LDH - Ht wk 5,Gills LDH 0.2954540850 0.2842454 544.24 1.039 0.2991

Ht wk 4,Gills LDH - Ht wk 5,Gills LDH -0.2215657453 0.2971732 532.10 -0.746 0.4563

Control,Glucose - Ht wk 0,Glucose -0.0964198253 0.3440727 536.48 -0.280 0.7794

Control,Glucose - Ht wk 1,Glucose -0.8788381741 0.2936041 544.51 -2.993 0.0029

Control,Glucose - Ht wk 2,Glucose -0.6217692730 0.2847912 546.55 -2.183 0.0294

Control,Glucose - Ht wk 3,Glucose -0.4436856997 0.2851883 542.48 -1.556 0.1203

Control,Glucose - Ht wk 4,Glucose -0.6121432771 0.2849073 544.27 -2.149 0.0321

Control,Glucose - Ht wk 5,Glucose -0.2124548736 0.2941828 539.95 -0.722 0.4705

Ht wk 0,Glucose - Ht wk 1,Glucose -0.7824183488 0.3435584 536.97 -2.277 0.0232

Ht wk 0,Glucose - Ht wk 2,Glucose -0.5253494477 0.3360582 539.27 -1.563 0.1186

Ht wk 0,Glucose - Ht wk 3,Glucose -0.3472658744 0.3363947 536.30 -1.032 0.3024

Ht wk 0,Glucose - Ht wk 4,Glucose -0.5157234519 0.3361565 536.66 -1.534 0.1256

Ht wk 0,Glucose - Ht wk 5,Glucose -0.1160350484 0.3440531 546.68 -0.337 0.7361

Ht wk 1,Glucose - Ht wk 2,Glucose 0.2570689011 0.2841698 544.82 0.905 0.3661

Ht wk 1,Glucose - Ht wk 3,Glucose 0.4351524744 0.2845677 545.23 1.529 0.1268

Ht wk 1,Glucose - Ht wk 4,Glucose 0.2666948969 0.2842861 544.65 0.938 0.3486

Ht wk 1,Glucose - Ht wk 5,Glucose 0.6663833004 0.2935812 534.28 2.270 0.0236

Ht wk 2,Glucose - Ht wk 3,Glucose 0.1780835733 0.2754659 546.98 0.646 0.5182

Ht wk 2,Glucose - Ht wk 4,Glucose 0.0096259958 0.2751750 546.71 0.035 0.9721

Ht wk 2,Glucose - Ht wk 5,Glucose 0.4093143993 0.2847676 528.77 1.437 0.1512

Ht wk 3,Glucose - Ht wk 4,Glucose -0.1684575775 0.2755859 543.51 -0.611 0.5413

Ht wk 3,Glucose - Ht wk 5,Glucose 0.2312308260 0.2851647 539.04 0.811 0.4178

Ht wk 4,Glucose - Ht wk 5,Glucose 0.3996884035 0.2848837 536.28 1.403 0.1612

Control,Hcrt (%) - Ht wk 0,Hcrt (%) -0.1348215888 0.2842660 542.96 -0.474 0.6355

Control,Hcrt (%) - Ht wk 1,Hcrt (%) -0.1327256182 0.3055438 540.91 -0.434 0.6642

Control,Hcrt (%) - Ht wk 2,Hcrt (%) -0.1056723365 0.2847912 546.55 -0.371 0.7107

Control,Hcrt (%) - Ht wk 3,Hcrt (%) -0.0768021770 0.2851883 542.48 -0.269 0.7878

Control,Hcrt (%) - Ht wk 4,Hcrt (%) -0.0131845289 0.2849073 544.27 -0.046 0.9631

Control,Hcrt (%) - Ht wk 5,Hcrt (%) 0.1969474876 0.3065559 535.28 0.642 0.5209

Ht wk 0,Hcrt (%) - Ht wk 1,Hcrt (%) 0.0020959706 0.2959852 539.33 0.007 0.9944

Ht wk 0,Hcrt (%) - Ht wk 2,Hcrt (%) 0.0291492523 0.2745109 545.44 0.106 0.9155

Ht wk 0,Hcrt (%) - Ht wk 3,Hcrt (%) 0.0580194119 0.2749229 542.52 0.211 0.8329

Ht wk 0,Hcrt (%) - Ht wk 4,Hcrt (%) 0.1216370599 0.2746314 543.55 0.443 0.6580

Ht wk 0,Hcrt (%) - Ht wk 5,Hcrt (%) 0.3317690764 0.2970299 532.41 1.117 0.2645

Ht wk 1,Hcrt (%) - Ht wk 2,Hcrt (%) 0.0270532817 0.2964896 542.96 0.091 0.9273

Ht wk 1,Hcrt (%) - Ht wk 3,Hcrt (%) 0.0559234412 0.2968711 541.13 0.188 0.8507

Ht wk 1,Hcrt (%) - Ht wk 4,Hcrt (%) 0.1195410893 0.2966011 541.18 0.403 0.6871

Ht wk 1,Hcrt (%) - Ht wk 5,Hcrt (%) 0.3296731058 0.3174533 538.54 1.038 0.2995

Ht wk 2,Hcrt (%) - Ht wk 3,Hcrt (%) 0.0288701596 0.2754659 546.98 0.105 0.9166

Ht wk 2,Hcrt (%) - Ht wk 4,Hcrt (%) 0.0924878076 0.2751750 546.71 0.336 0.7369

Ht wk 2,Hcrt (%) - Ht wk 5,Hcrt (%) 0.3026198241 0.2975326 523.40 1.017 0.3096

Ht wk 3,Hcrt (%) - Ht wk 4,Hcrt (%) 0.0636176480 0.2755859 543.51 0.231 0.8175

Ht wk 3,Hcrt (%) - Ht wk 5,Hcrt (%) 0.2737496645 0.2979127 533.51 0.919 0.3586

Ht wk 4,Hcrt (%) - Ht wk 5,Hcrt (%) 0.2101320165 0.2976437 530.99 0.706 0.4805

Control,Lactate - Ht wk 0,Lactate -0.2253865469 0.2842660 542.96 -0.793 0.4282

Control,Lactate - Ht wk 1,Lactate -0.2610766816 0.2936041 544.51 -0.889 0.3743

Control,Lactate - Ht wk 2,Lactate -0.1339864064 0.2847912 546.55 -0.470 0.6382

Control,Lactate - Ht wk 3,Lactate -0.1413337527 0.2947710 544.45 -0.479 0.6318

Control,Lactate - Ht wk 4,Lactate -0.1235771036 0.2849073 544.27 -0.434 0.6646

Control,Lactate - Ht wk 5,Lactate -0.1487685429 0.2941828 539.95 -0.506 0.6133

Ht wk 0,Lactate - Ht wk 1,Lactate -0.0356901347 0.2836434 543.02 -0.126 0.8999

Ht wk 0,Lactate - Ht wk 2,Lactate 0.0914001406 0.2745109 545.44 0.333 0.7393

Ht wk 0,Lactate - Ht wk 3,Lactate 0.0840527942 0.2848511 545.00 0.295 0.7680

Ht wk 0,Lactate - Ht wk 4,Lactate 0.1018094434 0.2746314 543.55 0.371 0.7110

Ht wk 0,Lactate - Ht wk 5,Lactate 0.0766180040 0.2842424 537.32 0.270 0.7876

Ht wk 1,Lactate - Ht wk 2,Lactate 0.1270902753 0.2841698 544.82 0.447 0.6549

Ht wk 1,Lactate - Ht wk 3,Lactate 0.1197429289 0.2941706 547.23 0.407 0.6841

Ht wk 1,Lactate - Ht wk 4,Lactate 0.1374995781 0.2842861 544.65 0.484 0.6288

Ht wk 1,Lactate - Ht wk 5,Lactate 0.1123081387 0.2935812 534.28 0.383 0.7022

Ht wk 2,Lactate - Ht wk 3,Lactate -0.0073473463 0.2853752 548.04 -0.026 0.9795

Ht wk 2,Lactate - Ht wk 4,Lactate 0.0104093028 0.2751750 546.71 0.038 0.9698

Ht wk 2,Lactate - Ht wk 5,Lactate -0.0147821365 0.2847676 528.77 -0.052 0.9586

Ht wk 3,Lactate - Ht wk 4,Lactate 0.0177566491 0.2854911 545.37 0.062 0.9504

Ht wk 3,Lactate - Ht wk 5,Lactate -0.0074347902 0.2947482 537.27 -0.025 0.9799

Ht wk 4,Lactate - Ht wk 5,Lactate -0.0251914394 0.2848837 536.28 -0.088 0.9296

Control,MCHC - Ht wk 0,MCHC -0.0026336202 0.2842660 542.96 -0.009 0.9926

Control,MCHC - Ht wk 1,MCHC -0.0764148009 0.3055438 540.91 -0.250 0.8026

Control,MCHC - Ht wk 2,MCHC -0.0789711126 0.2847912 546.55 -0.277 0.7817

Control,MCHC - Ht wk 3,MCHC -0.1229936193 0.2851883 542.48 -0.431 0.6664

Control,MCHC - Ht wk 4,MCHC -0.1787853546 0.2849073 544.27 -0.628 0.5306

Control,MCHC - Ht wk 5,MCHC -0.2706369228 0.3065559 535.28 -0.883 0.3777

Ht wk 0,MCHC - Ht wk 1,MCHC -0.0737811808 0.2959852 539.33 -0.249 0.8032

Ht wk 0,MCHC - Ht wk 2,MCHC -0.0763374924 0.2745109 545.44 -0.278 0.7811

Ht wk 0,MCHC - Ht wk 3,MCHC -0.1203599991 0.2749229 542.52 -0.438 0.6617

Ht wk 0,MCHC - Ht wk 4,MCHC -0.1761517345 0.2746314 543.55 -0.641 0.5215

Ht wk 0,MCHC - Ht wk 5,MCHC -0.2680033027 0.2970299 532.41 -0.902 0.3673

Ht wk 1,MCHC - Ht wk 2,MCHC -0.0025563117 0.2964896 542.96 -0.009 0.9931

Ht wk 1,MCHC - Ht wk 3,MCHC -0.0465788184 0.2968711 541.13 -0.157 0.8754

Ht wk 1,MCHC - Ht wk 4,MCHC -0.1023705537 0.2966011 541.18 -0.345 0.7301

Ht wk 1,MCHC - Ht wk 5,MCHC -0.1942221219 0.3174533 538.54 -0.612 0.5409

Ht wk 2,MCHC - Ht wk 3,MCHC -0.0440225067 0.2754659 546.98 -0.160 0.8731

Ht wk 2,MCHC - Ht wk 4,MCHC -0.0998142420 0.2751750 546.71 -0.363 0.7169

Ht wk 2,MCHC - Ht wk 5,MCHC -0.1916658102 0.2975326 523.40 -0.644 0.5197

Ht wk 3,MCHC - Ht wk 4,MCHC -0.0557917353 0.2755859 543.51 -0.202 0.8396

Ht wk 3,MCHC - Ht wk 5,MCHC -0.1476433035 0.2979127 533.51 -0.496 0.6204

Ht wk 4,MCHC - Ht wk 5,MCHC -0.0918515682 0.2976437 530.99 -0.309 0.7578

Control,Red Muscle CS - Ht wk 0,Red Muscle CS -0.0194818620 0.2747227 547.05 -0.071 0.9435

Control,Red Muscle CS - Ht wk 1,Red Muscle CS -0.1711082164 0.2746589 546.87 -0.623 0.5336

Control,Red Muscle CS - Ht wk 2,Red Muscle CS 0.0606030669 0.2752661 547.77 0.220 0.8258

Control,Red Muscle CS - Ht wk 3,Red Muscle CS 0.1315167359 0.2756769 546.89 0.477 0.6335

Control,Red Muscle CS - Ht wk 4,Red Muscle CS 0.7030162560 0.2753862 547.86 2.553 0.0110

Control,Red Muscle CS - Ht wk 5,Red Muscle CS 0.1848437705 0.2750863 531.97 0.672 0.5019

Ht wk 0,Red Muscle CS - Ht wk 1,Red Muscle CS -0.1516263544 0.2739021 543.52 -0.554 0.5801

Ht wk 0,Red Muscle CS - Ht wk 2,Red Muscle CS 0.0800849289 0.2745109 545.44 0.292 0.7706

Ht wk 0,Red Muscle CS - Ht wk 3,Red Muscle CS 0.1509985979 0.2749229 542.52 0.549 0.5831

Ht wk 0,Red Muscle CS - Ht wk 4,Red Muscle CS 0.7224981180 0.2746314 543.55 2.631 0.0088

Ht wk 0,Red Muscle CS - Ht wk 5,Red Muscle CS 0.2043256325 0.2743307 539.63 0.745 0.4567

Ht wk 1,Red Muscle CS - Ht wk 2,Red Muscle CS 0.2317112833 0.2744471 544.64 0.844 0.3989

Ht wk 1,Red Muscle CS - Ht wk 3,Red Muscle CS 0.3026249523 0.2748591 546.09 1.101 0.2714

Ht wk 1,Red Muscle CS - Ht wk 4,Red Muscle CS 0.8741244724 0.2745675 545.17 3.184 0.0015

Ht wk 1,Red Muscle CS - Ht wk 5,Red Muscle CS 0.3559519869 0.2742668 531.87 1.298 0.1949

Ht wk 2,Red Muscle CS - Ht wk 3,Red Muscle CS 0.0709136689 0.2754659 546.98 0.257 0.7969

Ht wk 2,Red Muscle CS - Ht wk 4,Red Muscle CS 0.6424131891 0.2751750 546.71 2.335 0.0199

Ht wk 2,Red Muscle CS - Ht wk 5,Red Muscle CS 0.1242407036 0.2748749 531.32 0.452 0.6515

Ht wk 3,Red Muscle CS - Ht wk 4,Red Muscle CS 0.5714995202 0.2755859 543.51 2.074 0.0386

Ht wk 3,Red Muscle CS - Ht wk 5,Red Muscle CS 0.0533270347 0.2752862 541.78 0.194 0.8465

Ht wk 4,Red Muscle CS - Ht wk 5,Red Muscle CS -0.5181724855 0.2749951 538.86 -1.884 0.0601

Control,Red Muscle LDH - Ht wk 0,Red Muscle LDH 0.8120095765 0.2747227 547.05 2.956 0.0033

Control,Red Muscle LDH - Ht wk 1,Red Muscle LDH 0.1956834701 0.2842452 546.14 0.688 0.4915

Control,Red Muscle LDH - Ht wk 2,Red Muscle LDH 0.5132131291 0.2851884 548.15 1.800 0.0725

Control,Red Muscle LDH - Ht wk 3,Red Muscle LDH -0.2935738811 0.2854066 548.12 -1.029 0.3041

Control,Red Muscle LDH - Ht wk 4,Red Muscle LDH -0.1556795269 0.2753862 547.86 -0.565 0.5721

Control,Red Muscle LDH - Ht wk 5,Red Muscle LDH -0.0986999366 0.2750863 531.97 -0.359 0.7199

Ht wk 0,Red Muscle LDH - Ht wk 1,Red Muscle LDH -0.6163261064 0.2835140 541.29 -2.174 0.0301

Ht wk 0,Red Muscle LDH - Ht wk 2,Red Muscle LDH -0.2987964474 0.2844596 546.59 -1.050 0.2940

Ht wk 0,Red Muscle LDH - Ht wk 3,Red Muscle LDH -1.1055834576 0.2846783 544.92 -3.884 0.0001

Ht wk 0,Red Muscle LDH - Ht wk 4,Red Muscle LDH -0.9676891034 0.2746314 543.55 -3.524 0.0005

Ht wk 0,Red Muscle LDH - Ht wk 5,Red Muscle LDH -0.9107095131 0.2743307 539.63 -3.320 0.0010

Ht wk 1,Red Muscle LDH - Ht wk 2,Red Muscle LDH 0.3175296590 0.2936666 545.31 1.081 0.2801

Ht wk 1,Red Muscle LDH - Ht wk 3,Red Muscle LDH -0.4892573511 0.2938784 545.20 -1.665 0.0965

Ht wk 1,Red Muscle LDH - Ht wk 4,Red Muscle LDH -0.3513629969 0.2841570 543.14 -1.237 0.2168

Ht wk 1,Red Muscle LDH - Ht wk 5,Red Muscle LDH -0.2943834067 0.2838663 538.20 -1.037 0.3002

Ht wk 2,Red Muscle LDH - Ht wk 3,Red Muscle LDH -0.8067870102 0.2947908 548.32 -2.737 0.0064

Ht wk 2,Red Muscle LDH - Ht wk 4,Red Muscle LDH -0.6688926560 0.2851005 547.54 -2.346 0.0193

Ht wk 2,Red Muscle LDH - Ht wk 5,Red Muscle LDH -0.6119130657 0.2848108 532.97 -2.148 0.0321

Ht wk 3,Red Muscle LDH - Ht wk 4,Red Muscle LDH 0.1378943542 0.2853187 545.79 0.483 0.6291

Ht wk 3,Red Muscle LDH - Ht wk 5,Red Muscle LDH 0.1948739445 0.2850293 540.79 0.684 0.4945

Ht wk 4,Red Muscle LDH - Ht wk 5,Red Muscle LDH 0.0569795903 0.2749951 538.86 0.207 0.8359

Control,Spleen [Hb] - Ht wk 0,Spleen [Hb] 0.1625157686 0.2747227 547.05 0.592 0.5544

Control,Spleen [Hb] - Ht wk 1,Spleen [Hb] -0.1016067369 0.2844252 547.17 -0.357 0.7211

Control,Spleen [Hb] - Ht wk 2,Spleen [Hb] -0.1136205417 0.2752661 547.77 -0.413 0.6799

Control,Spleen [Hb] - Ht wk 3,Spleen [Hb] -0.3566329769 0.2756769 546.89 -1.294 0.1963

Control,Spleen [Hb] - Ht wk 4,Spleen [Hb] -0.4197447929 0.2753862 547.86 -1.524 0.1280

Control,Spleen [Hb] - Ht wk 5,Spleen [Hb] -0.6044198407 0.2848789 534.30 -2.122 0.0343

Ht wk 0,Spleen [Hb] - Ht wk 1,Spleen [Hb] -0.2641225055 0.2836945 544.00 -0.931 0.3523

Ht wk 0,Spleen [Hb] - Ht wk 2,Spleen [Hb] -0.2761363103 0.2745109 545.44 -1.006 0.3149

Ht wk 0,Spleen [Hb] - Ht wk 3,Spleen [Hb] -0.5191487455 0.2749229 542.52 -1.888 0.0595

Ht wk 0,Spleen [Hb] - Ht wk 4,Spleen [Hb] -0.5822605615 0.2746314 543.55 -2.120 0.0344

Ht wk 0,Spleen [Hb] - Ht wk 5,Spleen [Hb] -0.7669356093 0.2841493 540.93 -2.699 0.0072

Ht wk 1,Spleen [Hb] - Ht wk 2,Spleen [Hb] -0.0120138048 0.2842208 545.22 -0.042 0.9663

Ht wk 1,Spleen [Hb] - Ht wk 3,Spleen [Hb] -0.2550262400 0.2846186 546.17 -0.896 0.3706

Ht wk 1,Spleen [Hb] - Ht wk 4,Spleen [Hb] -0.3181380560 0.2843371 545.48 -1.119 0.2637

Ht wk 1,Spleen [Hb] - Ht wk 5,Spleen [Hb] -0.5028131038 0.2935405 536.57 -1.713 0.0873

Ht wk 2,Spleen [Hb] - Ht wk 3,Spleen [Hb] -0.2430124352 0.2754659 546.98 -0.882 0.3781

Ht wk 2,Spleen [Hb] - Ht wk 4,Spleen [Hb] -0.3061242512 0.2751750 546.71 -1.112 0.2664

Ht wk 2,Spleen [Hb] - Ht wk 5,Spleen [Hb] -0.4907992990 0.2846748 533.69 -1.724 0.0853

Ht wk 3,Spleen [Hb] - Ht wk 4,Spleen [Hb] -0.0631118160 0.2755859 543.51 -0.229 0.8189

Ht wk 3,Spleen [Hb] - Ht wk 5,Spleen [Hb] -0.2477868638 0.2850720 542.88 -0.869 0.3851

Ht wk 4,Spleen [Hb] - Ht wk 5,Spleen [Hb] -0.1846750478 0.2847909 540.28 -0.648 0.5170

Control,SSI - Ht wk 0,SSI -0.0508899233 0.2747227 547.05 -0.185 0.8531

Control,SSI - Ht wk 1,SSI 0.0570791221 0.2746589 546.87 0.208 0.8354

Control,SSI - Ht wk 2,SSI 0.0059117482 0.2851165 548.03 0.021 0.9835

Control,SSI - Ht wk 3,SSI 0.1111853991 0.2756769 546.89 0.403 0.6869

Control,SSI - Ht wk 4,SSI -0.0467365890 0.2753862 547.86 -0.170 0.8653

Control,SSI - Ht wk 5,SSI -0.1520700146 0.2750863 531.97 -0.553 0.5806

Ht wk 0,SSI - Ht wk 1,SSI 0.1079690453 0.2739021 543.52 0.394 0.6936

Ht wk 0,SSI - Ht wk 2,SSI 0.0568016715 0.2843875 545.05 0.200 0.8418

Ht wk 0,SSI - Ht wk 3,SSI 0.1620753224 0.2749229 542.52 0.590 0.5558

Ht wk 0,SSI - Ht wk 4,SSI 0.0041533343 0.2746314 543.55 0.015 0.9879

Ht wk 0,SSI - Ht wk 5,SSI -0.1011800913 0.2743307 539.63 -0.369 0.7124

Ht wk 1,SSI - Ht wk 2,SSI -0.0511673738 0.2843259 545.20 -0.180 0.8573

Ht wk 1,SSI - Ht wk 3,SSI 0.0541062770 0.2748591 546.09 0.197 0.8440

Ht wk 1,SSI - Ht wk 4,SSI -0.1038157111 0.2745675 545.17 -0.378 0.7055

Ht wk 1,SSI - Ht wk 5,SSI -0.2091491367 0.2742668 531.87 -0.763 0.4461

Ht wk 2,SSI - Ht wk 3,SSI 0.1052736509 0.2853095 546.08 0.369 0.7123

Ht wk 2,SSI - Ht wk 4,SSI -0.0526483372 0.2850286 546.31 -0.185 0.8535

Ht wk 2,SSI - Ht wk 5,SSI -0.1579817628 0.2847388 536.61 -0.555 0.5792

Ht wk 3,SSI - Ht wk 4,SSI -0.1579219881 0.2755859 543.51 -0.573 0.5669

Ht wk 3,SSI - Ht wk 5,SSI -0.2632554137 0.2752862 541.78 -0.956 0.3393

Ht wk 4,SSI - Ht wk 5,SSI -0.1053334256 0.2749951 538.86 -0.383 0.7018

**Apogonid Blood Analyses**

***Model:*** Treatment*Tissue + (BM|Treatment:No)

***Overall Results***

|  | f | SumSq | MeanSq | Fvalue | upper.df | upper.p | lower.df | lower.p | expl.dev.(%) |
| --- | --- | --- | --- | --- | --- | --- | --- | --- | --- |
| Treatment | 6 | 4.1908 | 0.6985 | 1.9279 | 374 | 0.0753 | 250 | 0.0768 | 0.1368 |
| Tissue | 6 | 2886.3466 | 481.0578 | 1327.8173 | 374 | 0.0000 | 250 | 0.0000 | 94.2092 |
| Treatment : Tissue | 36 | 31.9333 | 0.8870 | 2.4484 | 374 | 0.0000 | 250 | 0.0000 | 1.0423 |

***Model mass adjusted least squares means (LN transformation)***

$lsmeans

Treatment Tissue lsmean SE df lower.CL upper.CL

Control Body condition 0.6527613 0.1534840 363.60 0.3509334 0.95458915

HT wk 0 Body condition 0.8081815 0.2428785 296.50 0.3301975 1.28616561

HT wk 1 Body condition 0.7249809 0.2173668 353.41 0.2974857 1.15247613

HT wk 2 Body condition 0.7196773 0.2168739 363.26 0.2931914 1.14616327

HT wk 3 Body condition 0.8168839 0.2162702 361.90 0.3915799 1.24218800

HT wk 4 Body condition 0.6981464 0.2151692 329.43 0.2748675 1.12142532

HT wk 5 Body condition 0.8115225 0.2477953 227.09 0.3232505 1.29979453

Control Gills CS 3.8497232 0.1582256 365.46 3.5385762 4.16087019

HT wk 0 Gills CS 4.1528583 0.2263121 299.99 3.7074980 4.59821866

HT wk 1 Gills CS 3.9511616 0.2318618 361.14 3.4951927 4.40713047

HT wk 2 Gills CS 3.8089718 0.2168739 363.26 3.3824859 4.23545774

HT wk 3 Gills CS 4.1404002 0.2315537 365.74 3.6850564 4.59574401

HT wk 4 Gills CS 3.8249554 0.2151692 329.43 3.4016765 4.24823427

HT wk 5 Gills CS 3.5931105 0.2477953 227.09 3.1048385 4.08138255

Control Gills LDH 5.7752235 0.1534840 363.60 5.4733956 6.07705136

HT wk 0 Gills LDH 5.7796115 0.2263121 299.99 5.3342512 6.22497185

HT wk 1 Gills LDH 6.1367850 0.2173668 353.41 5.7092898 6.56428022

HT wk 2 Gills LDH 5.7432175 0.2168739 363.26 5.3167316 6.16970347

HT wk 3 Gills LDH 5.5282554 0.2315537 365.74 5.0729116 5.98359920

HT wk 4 Gills LDH 5.9178040 0.2151692 329.43 5.4945251 6.34108289

HT wk 5 Gills LDH 5.7730028 0.2477953 227.09 5.2847308 6.26127484

Control Red Muscle CS 4.7500233 0.1584764 365.32 4.4383827 5.06166384

HT wk 0 Red Muscle CS 4.7289701 0.2263121 299.99 4.2836098 5.17433039

HT wk 1 Red Muscle CS 5.1541425 0.2173668 353.41 4.7266473 5.58163772

HT wk 2 Red Muscle CS 4.9678582 0.2321136 364.02 4.5114063 5.42431014

HT wk 3 Red Muscle CS 5.2625456 0.2162702 361.90 4.8372416 5.68784970

HT wk 4 Red Muscle CS 5.5186509 0.2151692 329.43 5.0953720 5.94192978

HT wk 5 Red Muscle CS 5.2999164 0.2477953 227.09 4.8116444 5.78818841

Control Red Muscle LDH 6.0165439 0.1534840 363.60 5.7147160 6.31837171

HT wk 0 Red Muscle LDH 6.1531812 0.2331298 337.12 5.6946089 6.61175352

HT wk 1 Red Muscle LDH 6.3634113 0.2173668 353.41 5.9359161 6.79090649

HT wk 2 Red Muscle LDH 5.9458627 0.2168739 363.26 5.5193767 6.37234864

HT wk 3 Red Muscle LDH 6.6297198 0.2162702 361.90 6.2044158 7.05502386

HT wk 4 Red Muscle LDH 6.1216752 0.2151692 329.43 5.6983963 6.54495415

HT wk 5 Red Muscle LDH 5.3095178 0.2477953 227.09 4.8212458 5.79778978

Control Spleen [Hb] 1.3149075 0.1534840 363.60 1.0130797 1.61673540

HT wk 0 Spleen [Hb] 1.3179398 0.2263121 299.99 0.8725795 1.76330013

HT wk 1 Spleen [Hb] 1.3662950 0.2173668 353.41 0.9387998 1.79379016

HT wk 2 Spleen [Hb] 1.6975317 0.2168739 363.26 1.2710458 2.12401767

HT wk 3 Spleen [Hb] 1.4403111 0.2315057 366.64 0.9850654 1.89555676

HT wk 4 Spleen [Hb] 1.2781548 0.2151692 329.43 0.8548759 1.70143374

HT wk 5 Spleen [Hb] 1.2365867 0.3040275 259.86 0.6379154 1.83525798

Control SSI -0.7667496 0.1534840 363.60 -1.0685775 -0.46492179

HT wk 0 SSI -0.7663151 0.2263121 299.99 -1.2116754 -0.32095473

HT wk 1 SSI -0.3905764 0.2173668 353.41 -0.8180716 0.03691883

HT wk 2 SSI -0.8369532 0.2168739 363.26 -1.2634391 -0.41046722

HT wk 3 SSI -1.6633497 0.2162702 361.90 -2.0886538 -1.23804567

HT wk 4 SSI -1.7894684 0.2151692 329.43 -2.2127473 -1.36618948

HT wk 5 SSI -2.3318742 0.2477953 227.09 -2.8201462 -1.84360215

***Planned Contrast analyses using FDR correction for Type II error (Pcutoff = 0.0262)***

$contrasts

contrast estimate SE df t.ratio p.value

Control,Body condition - HT wk 0,Body condition -0.1554202534 0.2873104 331.51 -0.541 0.5889

Control,Body condition - HT wk 1,Body condition -0.0722196489 0.2660934 357.50 -0.271 0.7862

Control,Body condition - HT wk 2,Body condition -0.0669160376 0.2656908 363.39 -0.252 0.8013

Control,Body condition - HT wk 3,Body condition -0.1641226550 0.2651983 366.60 -0.619 0.5364

Control,Body condition - HT wk 4,Body condition -0.0453851227 0.2643012 355.59 -0.172 0.8638

Control,Body condition - HT wk 5,Body condition -0.1587612316 0.2914787 289.40 -0.545 0.5864

HT wk 0,Body condition - HT wk 1,Body condition 0.0832006044 0.3259422 338.84 0.255 0.7987

HT wk 0,Body condition - HT wk 2,Body condition 0.0885042158 0.3256136 347.86 0.272 0.7859

HT wk 0,Body condition - HT wk 3,Body condition -0.0087024016 0.3252118 359.19 -0.027 0.9787

HT wk 0,Body condition - HT wk 4,Body condition 0.1100351307 0.3244807 362.38 0.339 0.7347

HT wk 0,Body condition - HT wk 5,Body condition -0.0033409782 0.3469761 309.81 -0.010 0.9923

HT wk 1,Body condition - HT wk 2,Body condition 0.0053036114 0.3070548 359.35 0.017 0.9862

HT wk 1,Body condition - HT wk 3,Body condition -0.0919030061 0.3066286 367.07 -0.300 0.7646

HT wk 1,Body condition - HT wk 4,Body condition 0.0268345262 0.3058531 367.49 0.088 0.9301

HT wk 1,Body condition - HT wk 5,Body condition -0.0865415826 0.3296223 331.72 -0.263 0.7931

HT wk 2,Body condition - HT wk 3,Body condition -0.0972066174 0.3062794 366.80 -0.317 0.7511

HT wk 2,Body condition - HT wk 4,Body condition 0.0215309149 0.3055030 362.78 0.070 0.9439

HT wk 2,Body condition - HT wk 5,Body condition -0.0918451940 0.3292974 323.72 -0.279 0.7805

HT wk 3,Body condition - HT wk 4,Body condition 0.1187375323 0.3050747 349.43 0.389 0.6974

HT wk 3,Body condition - HT wk 5,Body condition 0.0053614234 0.3289001 303.69 0.016 0.9870

HT wk 4,Body condition - HT wk 5,Body condition -0.1133761089 0.3281772 279.63 -0.345 0.7300

Control,Gills CS - HT wk 0,Gills CS -0.3031351441 0.2761386 339.61 -1.098 0.2731

Control,Gills CS - HT wk 1,Gills CS -0.1014383950 0.2807049 362.89 -0.361 0.7180

Control,Gills CS - HT wk 2,Gills CS 0.0407513976 0.2684579 364.12 0.152 0.8794

Control,Gills CS - HT wk 3,Gills CS -0.2906770254 0.2804505 367.46 -1.036 0.3007

Control,Gills CS - HT wk 4,Gills CS 0.0247678357 0.2670826 354.13 0.093 0.9262

Control,Gills CS - HT wk 5,Gills CS 0.2566126557 0.2940031 290.15 0.873 0.3835

HT wk 0,Gills CS - HT wk 1,Gills CS 0.2016967490 0.3240017 349.92 0.623 0.5340

HT wk 0,Gills CS - HT wk 2,Gills CS 0.3438865417 0.3134509 350.25 1.097 0.2734

HT wk 0,Gills CS - HT wk 3,Gills CS 0.0124581187 0.3237813 363.54 0.038 0.9693

HT wk 0,Gills CS - HT wk 4,Gills CS 0.3279029798 0.3122738 366.02 1.050 0.2944

HT wk 0,Gills CS - HT wk 5,Gills CS 0.5597477998 0.3355885 310.45 1.668 0.0963

HT wk 1,Gills CS - HT wk 2,Gills CS 0.1421897927 0.3174810 362.27 0.448 0.6545

HT wk 1,Gills CS - HT wk 3,Gills CS -0.1892386303 0.3276844 367.83 -0.578 0.5640

HT wk 1,Gills CS - HT wk 4,Gills CS 0.1262062308 0.3163190 365.81 0.399 0.6901

HT wk 1,Gills CS - HT wk 5,Gills CS 0.3580510507 0.3393559 333.69 1.055 0.2921

HT wk 2,Gills CS - HT wk 3,Gills CS -0.3314284230 0.3172561 367.67 -1.045 0.2969

HT wk 2,Gills CS - HT wk 4,Gills CS -0.0159835619 0.3055030 362.78 -0.052 0.9583

HT wk 2,Gills CS - HT wk 5,Gills CS 0.2158612581 0.3292974 323.72 0.656 0.5126

HT wk 3,Gills CS - HT wk 4,Gills CS 0.3154448611 0.3160932 353.75 0.998 0.3190

HT wk 3,Gills CS - HT wk 5,Gills CS 0.5472896810 0.3391454 312.29 1.614 0.1076

HT wk 4,Gills CS - HT wk 5,Gills CS 0.2318448199 0.3281772 279.63 0.706 0.4805

Control,Gills LDH - HT wk 0,Gills LDH -0.0043880322 0.2734493 335.26 -0.016 0.9872

Control,Gills LDH - HT wk 1,Gills LDH -0.3615615263 0.2660934 357.50 -1.359 0.1751

Control,Gills LDH - HT wk 2,Gills LDH 0.0320059713 0.2656908 363.39 0.120 0.9042

Control,Gills LDH - HT wk 3,Gills LDH 0.2469680869 0.2778030 368.01 0.889 0.3746

Control,Gills LDH - HT wk 4,Gills LDH -0.1425804853 0.2643012 355.59 -0.539 0.5899

Control,Gills LDH - HT wk 5,Gills LDH 0.0022206623 0.2914787 289.40 0.008 0.9939

HT wk 0,Gills LDH - HT wk 1,Gills LDH -0.3571734941 0.3137922 340.57 -1.138 0.2558

HT wk 0,Gills LDH - HT wk 2,Gills LDH 0.0363940035 0.3134509 350.25 0.116 0.9076

HT wk 0,Gills LDH - HT wk 3,Gills LDH 0.2513561190 0.3237813 363.54 0.776 0.4381

HT wk 0,Gills LDH - HT wk 4,Gills LDH -0.1381924532 0.3122738 366.02 -0.443 0.6584

HT wk 0,Gills LDH - HT wk 5,Gills LDH 0.0066086944 0.3355885 310.45 0.020 0.9843

HT wk 1,Gills LDH - HT wk 2,Gills LDH 0.3935674976 0.3070548 359.35 1.282 0.2008

HT wk 1,Gills LDH - HT wk 3,Gills LDH 0.6085296132 0.3175933 367.57 1.916 0.0561

HT wk 1,Gills LDH - HT wk 4,Gills LDH 0.2189810410 0.3058531 367.49 0.716 0.4745

HT wk 1,Gills LDH - HT wk 5,Gills LDH 0.3637821886 0.3296223 331.72 1.104 0.2706

HT wk 2,Gills LDH - HT wk 3,Gills LDH 0.2149621156 0.3172561 367.67 0.678 0.4985

HT wk 2,Gills LDH - HT wk 4,Gills LDH -0.1745864566 0.3055030 362.78 -0.571 0.5680

HT wk 2,Gills LDH - HT wk 5,Gills LDH -0.0297853090 0.3292974 323.72 -0.090 0.9280

HT wk 3,Gills LDH - HT wk 4,Gills LDH -0.3895485722 0.3160932 353.75 -1.232 0.2186

HT wk 3,Gills LDH - HT wk 5,Gills LDH -0.2447474246 0.3391454 312.29 -0.722 0.4710

HT wk 4,Gills LDH - HT wk 5,Gills LDH 0.1448011476 0.3281772 279.63 0.441 0.6594

Control,Red Muscle CS - HT wk 0,Red Muscle CS 0.0210531987 0.2762824 337.97 0.076 0.9393

Control,Red Muscle CS - HT wk 1,Red Muscle CS -0.4041192611 0.2690040 358.63 -1.502 0.1339

Control,Red Muscle CS - HT wk 2,Red Muscle CS -0.2178349313 0.2810543 364.49 -0.775 0.4388

Control,Red Muscle CS - HT wk 3,Red Muscle CS -0.5125223743 0.2681186 366.53 -1.912 0.0567

Control,Red Muscle CS - HT wk 4,Red Muscle CS -0.7686276020 0.2672313 355.49 -2.876 0.0043

Control,Red Muscle CS - HT wk 5,Red Muscle CS -0.5498931308 0.2941382 291.14 -1.870 0.0626

HT wk 0,Red Muscle CS - HT wk 1,Red Muscle CS -0.4251724598 0.3137922 340.57 -1.355 0.1763

HT wk 0,Red Muscle CS - HT wk 2,Red Muscle CS -0.2388881300 0.3241819 351.45 -0.737 0.4617

HT wk 0,Red Muscle CS - HT wk 3,Red Muscle CS -0.5335755730 0.3130335 362.63 -1.705 0.0891

HT wk 0,Red Muscle CS - HT wk 4,Red Muscle CS -0.7896808007 0.3122738 366.02 -2.529 0.0119

HT wk 0,Red Muscle CS - HT wk 5,Red Muscle CS -0.5709463295 0.3355885 310.45 -1.701 0.0899

HT wk 1,Red Muscle CS - HT wk 2,Red Muscle CS 0.1862843298 0.3180017 359.83 0.586 0.5584

HT wk 1,Red Muscle CS - HT wk 3,Red Muscle CS -0.1084031132 0.3066286 367.07 -0.354 0.7239

HT wk 1,Red Muscle CS - HT wk 4,Red Muscle CS -0.3645083409 0.3058531 367.49 -1.192 0.2341

HT wk 1,Red Muscle CS - HT wk 5,Red Muscle CS -0.1457738698 0.3296223 331.72 -0.442 0.6586

HT wk 2,Red Muscle CS - HT wk 3,Red Muscle CS -0.2946874430 0.3172531 367.82 -0.929 0.3536

HT wk 2,Red Muscle CS - HT wk 4,Red Muscle CS -0.5507926707 0.3165036 365.38 -1.740 0.0827

HT wk 2,Red Muscle CS - HT wk 5,Red Muscle CS -0.3320581995 0.3395279 331.27 -0.978 0.3288

HT wk 3,Red Muscle CS - HT wk 4,Red Muscle CS -0.2561052277 0.3050747 349.43 -0.839 0.4018

HT wk 3,Red Muscle CS - HT wk 5,Red Muscle CS -0.0373707566 0.3289001 303.69 -0.114 0.9096

HT wk 4,Red Muscle CS - HT wk 5,Red Muscle CS 0.2187344711 0.3281772 279.63 0.667 0.5056

Control,Red Muscle LDH - HT wk 0,Red Muscle LDH -0.1366373500 0.2791180 348.04 -0.490 0.6248

Control,Red Muscle LDH - HT wk 1,Red Muscle LDH -0.3468674452 0.2660934 357.50 -1.304 0.1932

Control,Red Muscle LDH - HT wk 2,Red Muscle LDH 0.0706811610 0.2656908 363.39 0.266 0.7904

Control,Red Muscle LDH - HT wk 3,Red Muscle LDH -0.6131759537 0.2651983 366.60 -2.312 0.0213

Control,Red Muscle LDH - HT wk 4,Red Muscle LDH -0.1051313899 0.2643012 355.59 -0.398 0.6910

Control,Red Muscle LDH - HT wk 5,Red Muscle LDH 0.7070260787 0.2914787 289.40 2.426 0.0159

HT wk 1,Red Muscle LDH - HT wk 2,Red Muscle LDH 0.4175486062 0.3070548 359.35 1.360 0.1747

HT wk 1,Red Muscle LDH - HT wk 3,Red Muscle LDH -0.2663085085 0.3066286 367.07 -0.869 0.3857

HT wk 1,Red Muscle LDH - HT wk 4,Red Muscle LDH 0.2417360553 0.3058531 367.49 0.790 0.4298

HT wk 1,Red Muscle LDH - HT wk 5,Red Muscle LDH 1.0538935239 0.3296223 331.72 3.197 0.0015

HT wk 2,Red Muscle LDH - HT wk 3,Red Muscle LDH -0.6838571147 0.3062794 366.80 -2.233 0.0262

HT wk 2,Red Muscle LDH - HT wk 4,Red Muscle LDH -0.1758125509 0.3055030 362.78 -0.575 0.5653

HT wk 2,Red Muscle LDH - HT wk 5,Red Muscle LDH 0.6363449177 0.3292974 323.72 1.932 0.0542

HT wk 3,Red Muscle LDH - HT wk 4,Red Muscle LDH 0.5080445638 0.3050747 349.43 1.665 0.0967

HT wk 3,Red Muscle LDH - HT wk 5,Red Muscle LDH 1.3202020324 0.3289001 303.69 4.014 0.0001

HT wk 4,Red Muscle LDH - HT wk 5,Red Muscle LDH 0.8121574686 0.3281772 279.63 2.475 0.0139

Control,Spleen [Hb] - HT wk 0,Spleen [Hb] -0.0030322696 0.2734493 335.26 -0.011 0.9912

Control,Spleen [Hb] - HT wk 1,Spleen [Hb] -0.0513874324 0.2660934 357.50 -0.193 0.8470

Control,Spleen [Hb] - HT wk 2,Spleen [Hb] -0.3826241870 0.2656908 363.39 -1.440 0.1507

Control,Spleen [Hb] - HT wk 3,Spleen [Hb] -0.1254035515 0.2777629 367.60 -0.451 0.6519

Control,Spleen [Hb] - HT wk 4,Spleen [Hb] 0.0367527040 0.2643012 355.59 0.139 0.8895

Control,Spleen [Hb] - HT wk 5,Spleen [Hb] 0.0783208411 0.3405732 298.60 0.230 0.8183

HT wk 0,Spleen [Hb] - HT wk 1,Spleen [Hb] -0.0483551628 0.3137922 340.57 -0.154 0.8776

HT wk 0,Spleen [Hb] - HT wk 2,Spleen [Hb] -0.3795919174 0.3134509 350.25 -1.211 0.2267

HT wk 0,Spleen [Hb] - HT wk 3,Spleen [Hb] -0.1223712819 0.3237469 362.24 -0.378 0.7057

HT wk 0,Spleen [Hb] - HT wk 4,Spleen [Hb] 0.0397849736 0.3122738 366.02 0.127 0.8987

HT wk 0,Spleen [Hb] - HT wk 5,Spleen [Hb] 0.0813531107 0.3790118 328.21 0.215 0.8302

HT wk 1,Spleen [Hb] - HT wk 2,Spleen [Hb] -0.3312367546 0.3070548 359.35 -1.079 0.2814

HT wk 1,Spleen [Hb] - HT wk 3,Spleen [Hb] -0.0740161191 0.3175583 366.16 -0.233 0.8158

HT wk 1,Spleen [Hb] - HT wk 4,Spleen [Hb] 0.0881401363 0.3058531 367.49 0.288 0.7734

HT wk 1,Spleen [Hb] - HT wk 5,Spleen [Hb] 0.1297082735 0.3737393 330.21 0.347 0.7288

HT wk 2,Spleen [Hb] - HT wk 3,Spleen [Hb] 0.2572206355 0.3172210 366.97 0.811 0.4180

HT wk 2,Spleen [Hb] - HT wk 4,Spleen [Hb] 0.4193768910 0.3055030 362.78 1.373 0.1707

HT wk 2,Spleen [Hb] - HT wk 5,Spleen [Hb] 0.4609450281 0.3734528 322.28 1.234 0.2180

HT wk 3,Spleen [Hb] - HT wk 4,Spleen [Hb] 0.1621562555 0.3160580 356.15 0.513 0.6082

HT wk 3,Spleen [Hb] - HT wk 5,Spleen [Hb] 0.2037243926 0.3821356 315.48 0.533 0.5943

HT wk 4,Spleen [Hb] - HT wk 5,Spleen [Hb] 0.0415681371 0.3724655 286.20 0.112 0.9112

Control,SSI - HT wk 0,SSI -0.0004345993 0.2734493 335.26 -0.002 0.9987

Control,SSI - HT wk 1,SSI -0.3761732901 0.2660934 357.50 -1.414 0.1583

Control,SSI - HT wk 2,SSI 0.0702035147 0.2656908 363.39 0.264 0.7918

Control,SSI - HT wk 3,SSI 0.8966000791 0.2651983 366.60 3.381 0.0008

Control,SSI - HT wk 4,SSI 1.0227187392 0.2643012 355.59 3.870 0.0001

Control,SSI - HT wk 5,SSI 1.5651245067 0.2914787 289.40 5.370 <.0001

HT wk 0,SSI - HT wk 1,SSI -0.3757386908 0.3137922 340.57 -1.197 0.2320

HT wk 0,SSI - HT wk 2,SSI 0.0706381141 0.3134509 350.25 0.225 0.8218

HT wk 0,SSI - HT wk 3,SSI 0.8970346784 0.3130335 362.63 2.866 0.0044

HT wk 0,SSI - HT wk 4,SSI 1.0231533386 0.3122738 366.02 3.276 0.0012

HT wk 0,SSI - HT wk 5,SSI 1.5655591060 0.3355885 310.45 4.665 <.0001

HT wk 1,SSI - HT wk 2,SSI 0.4463768049 0.3070548 359.35 1.454 0.1469

HT wk 1,SSI - HT wk 3,SSI 1.2727733692 0.3066286 367.07 4.151 <.0001

HT wk 1,SSI - HT wk 4,SSI 1.3988920293 0.3058531 367.49 4.574 <.0001

HT wk 1,SSI - HT wk 5,SSI 1.9412977968 0.3296223 331.72 5.889 <.0001

HT wk 2,SSI - HT wk 3,SSI 0.8263965643 0.3062794 366.80 2.698 0.0073

HT wk 2,SSI - HT wk 4,SSI 0.9525152245 0.3055030 362.78 3.118 0.0020

HT wk 2,SSI - HT wk 5,SSI 1.4949209919 0.3292974 323.72 4.540 <.0001

HT wk 3,SSI - HT wk 4,SSI 0.1261186602 0.3050747 349.43 0.413 0.6796

HT wk 3,SSI - HT wk 5,SSI 0.6685244276 0.3289001 303.69 2.033 0.0430

HT wk 4,SSI - HT wk 5,SSI 0.5424057674 0.3281772 279.63 1.653 0.0995

**Fusilera Gill Analyses**

***Model:*** Treatment*Measurement+(1|Treatment:ID:Loci.gill:Filament:Loci.fil)

***Overall Results:***

|  | f | SumSq | MeanSq | Fvalue | upper.df | upper.p | lower.df | lower.p | expl.dev.(%) |
| --- | --- | --- | --- | --- | --- | --- | --- | --- | --- |
| Treatment | 6 | 0.0887 | 0.0148 | 36.455 | 5696 | 0 | 5090 | 0 | 0.0561 |
| Measurement | 2 | 126.7193 | 63.3596 | 156237.1 | 5696 | 0 | 5090 | 0 | 80.1803 |
| Treatment : Measurement | 12 | 0.0374 | 0.0031 | 7.6854 | 5696 | 0 | 5090 | 0 | 0.0237 |

***Least squares means (Coxbox exponent = -0.15)***

| Treatment | Measurement | N | lsmean | SE |
| --- | --- | --- | --- | --- |
| Control | Ep.thick | 12 | 1.027764 | 0.004241 |
| Control | Lam.per | 10 | 0.464175 | 0.00126 |
| Control | Width | 10 | 0.747349 | 0.00126 |
| HT wk 0 | Ep.thick | 8 | 1.041248 | 0.004684 |
| HT wk 0 | Lam.per | 8 | 0.462392 | 0.001382 |
| HT wk 0 | Width | 8 | 0.748364 | 0.001395 |
| HT wk 1 | Ep.thick | 7 | 1.035416 | 0.005513 |
| HT wk 1 | Lam.per | 5 | 0.461911 | 0.002224 |
| HT wk 1 | Width | 5 | 0.735567 | 0.002293 |
| HT wk 2 | Ep.thick | 5 | 1.01133 | 0.006956 |
| HT wk 2 | Lam.per | 5 | 0.461584 | 0.001829 |
| HT wk 2 | Width | 5 | 0.734283 | 0.001847 |
| HT wk 3 | Ep.thick | 8 | 1.023461 | 0.00492 |
| HT wk 3 | Lam.per | 5 | 0.447651 | 0.001727 |
| HT wk 3 | Width | 5 | 0.731608 | 0.001744 |
| HT wk 4 | Ep.thick | 7 | 1.040813 | 0.005547 |
| HT wk 4 | Lam.per | 5 | 0.455236 | 0.001936 |
| HT wk 4 | Width | 5 | 0.741141 | 0.001936 |
| HT wk 5 | Ep.thick | 6 | 1.025107 | 0.005954 |
| HT wk 5 | Lam.per | 6 | 0.460144 | 0.001663 |
| HT wk 5 | Width | 6 | 0.745927 | 0.001662 |

***Planned contrast analyses using FDR correction for Type II error (Pcutoff = 0.001260)***

|  | Treatment1 | Measurement1 | Treatment.2 | Measurement.2 | p.value | sig2 |
| --- | --- | --- | --- | --- | --- | --- |
| 1 | Control | Ep.thick | HT wk 0 | Ep.thick | 0.857549 | No |
| 2 | Control | Ep.thick | HT wk 1 | Ep.thick | 0.999961 | No |
| 3 | Control | Ep.thick | HT wk 2 | Ep.thick | 0.910233 | No |
| 4 | Control | Ep.thick | HT wk 3 | Ep.thick | 1 | No |
| 5 | Control | Ep.thick | HT wk 4 | Ep.thick | 0.955799 | No |
| 6 | Control | Ep.thick | HT wk 5 | Ep.thick | 1 | No |
| 21 | HT wk 0 | Ep.thick | HT wk 1 | Ep.thick | 1 | No |
| 22 | HT wk 0 | Ep.thick | HT wk 2 | Ep.thick | 0.050267 | No |
| 23 | HT wk 0 | Ep.thick | HT wk 3 | Ep.thick | 0.514889 | No |
| 24 | HT wk 0 | Ep.thick | HT wk 4 | Ep.thick | 1 | No |
| 25 | HT wk 0 | Ep.thick | HT wk 5 | Ep.thick | 0.859302 | No |
| 40 | HT wk 1 | Ep.thick | HT wk 2 | Ep.thick | 0.440454 | No |
| 41 | HT wk 1 | Ep.thick | HT wk 3 | Ep.thick | 0.990862 | No |
| 42 | HT wk 1 | Ep.thick | HT wk 4 | Ep.thick | 1 | No |
| 43 | HT wk 1 | Ep.thick | HT wk 5 | Ep.thick | 0.999642 | No |
| 58 | HT wk 2 | Ep.thick | HT wk 3 | Ep.thick | 0.998213 | No |
| 59 | HT wk 2 | Ep.thick | HT wk 4 | Ep.thick | 0.109701 | No |
| 60 | HT wk 2 | Ep.thick | HT wk 5 | Ep.thick | 0.996293 | No |
| 75 | HT wk 3 | Ep.thick | HT wk 4 | Ep.thick | 0.729684 | No |
| 76 | HT wk 3 | Ep.thick | HT wk 5 | Ep.thick | 1 | No |
| 91 | HT wk 4 | Ep.thick | HT wk 5 | Ep.thick | 0.939727 | No |
| 120 | Control | Lam.per | HT wk 0 | Lam.per | 0.999996 | No |
| 121 | Control | Lam.per | HT wk 1 | Lam.per | 0.999999 | No |
| 122 | Control | Lam.per | HT wk 2 | Lam.per | 0.999901 | No |
| 123 | Control | Lam.per | HT wk 3 | Lam.per | 2.41E-12 | Yes |
| 124 | Control | Lam.per | HT wk 4 | Lam.per | 0.017435 | No |
| 125 | Control | Lam.per | HT wk 5 | Lam.per | 0.939146 | No |
| 133 | HT wk 0 | Lam.per | HT wk 1 | Lam.per | 1 | No |
| 134 | HT wk 0 | Lam.per | HT wk 2 | Lam.per | 1 | No |
| 135 | HT wk 0 | Lam.per | HT wk 3 | Lam.per | 5.58E-09 | Yes |
| 136 | HT wk 0 | Lam.per | HT wk 4 | Lam.per | 0.242384 | No |
| 137 | HT wk 0 | Lam.per | HT wk 5 | Lam.per | 0.999984 | No |
| 145 | HT wk 1 | Lam.per | HT wk 2 | Lam.per | 1 | No |
| 146 | HT wk 1 | Lam.per | HT wk 3 | Lam.per | 8.26E-05 | Yes |
| 147 | HT wk 1 | Lam.per | HT wk 4 | Lam.per | 0.781727 | No |
| 148 | HT wk 1 | Lam.per | HT wk 5 | Lam.per | 1 | No |
| 156 | HT wk 2 | Lam.per | HT wk 3 | Lam.per | 6.28E-06 | Yes |
| 157 | HT wk 2 | Lam.per | HT wk 4 | Lam.per | 0.698215 | No |
| 158 | HT wk 2 | Lam.per | HT wk 5 | Lam.per | 1 | No |
| 166 | HT wk 3 | Lam.per | HT wk 4 | Lam.per | 0.292934 | No |
| 167 | HT wk 3 | Lam.per | HT wk 5 | Lam.per | 3.83E-05 | Yes |
| 175 | HT wk 4 | Lam.per | HT wk 5 | Lam.per | 0.941748 | No |
| 190 | Control | Width | HT wk 0 | Width | 1 | No |
| 191 | Control | Width | HT wk 1 | Width | 0.001263 | Yes |
| 192 | Control | Width | HT wk 2 | Width | 1.07E-06 | Yes |
| 193 | Control | Width | HT wk 3 | Width | 5.34E-11 | Yes |
| 194 | Control | Width | HT wk 4 | Width | 0.460857 | No |
| 195 | Control | Width | HT wk 5 | Width | 1 | No |
| 196 | HT wk 0 | Width | HT wk 1 | Width | 0.000364 | Yes |
| 197 | HT wk 0 | Width | HT wk 2 | Width | 2.46E-07 | Yes |
| 198 | HT wk 0 | Width | HT wk 3 | Width | 1.3E-11 | Yes |
| 199 | HT wk 0 | Width | HT wk 4 | Width | 0.232024 | No |
| 200 | HT wk 0 | Width | HT wk 5 | Width | 0.999945 | No |
| 201 | HT wk 1 | Width | HT wk 2 | Width | 1 | No |
| 202 | HT wk 1 | Width | HT wk 3 | Width | 0.998896 | No |
| 203 | HT wk 1 | Width | HT wk 4 | Width | 0.958379 | No |
| 204 | HT wk 1 | Width | HT wk 5 | Width | 0.037099 | No |
| 205 | HT wk 2 | Width | HT wk 3 | Width | 0.99998 | No |
| 206 | HT wk 2 | Width | HT wk 4 | Width | 0.558961 | No |
| 207 | HT wk 2 | Width | HT wk 5 | Width | 0.000541 | Yes |
| 208 | HT wk 3 | Width | HT wk 4 | Width | 0.03698 | No |
| 209 | HT wk 3 | Width | HT wk 5 | Width | 5.79E-07 | Yes |
| 210 | HT wk 4 | Width | HT wk 5 | Width | 0.954231 | No |

**Apogonid Gill Analyses**

***Model:*** Treatment*Measurement+(1|Treatment:ID:Loci.gill:Filament:Loci.fil)

***Overall results:***

|  | f | SumSq | MeanSq | Fvalue | upper.df | upper.p | lower.df | lower.p | expl.dev.(%) |
| --- | --- | --- | --- | --- | --- | --- | --- | --- | --- |
| Treatment | 5 | 0.1191 | 0.0238 | 97.6513 | 2305 | 0 | 2043 | 0 | 0.2859 |
| Measurement | 2 | 31.9537 | 15.9768 | 65475.07 | 2305 | 0 | 2043 | 0 | 76.6745 |
| Treatment : Measurement | 10 | 0.0283 | 0.0028 | 11.6128 | 2305 | 0 | 2043 | 0 | 0.068 |

***Least squares means (Coxbox exponent = -0.09)***

| Treatment | Measurement | N | lsmean | SE |
| --- | --- | --- | --- | --- |
| Control | Ep.thick | 7 | 1.023816 | 0.003059 |
| Control | Lam.per | 6 | 0.625089 | 0.001444 |
| Control | Width | 6 | 0.836286 | 0.001449 |
| HT wk 0 | Ep.thick | 5 | 1.017416 | 0.004515 |
| HT wk 0 | Lam.per | 5 | 0.620562 | 0.001726 |
| HT wk 0 | Width | 5 | 0.836935 | 0.001731 |
| HT wk 1 | Ep.thick | 5 | 1.030257 | 0.004515 |
| HT wk 1 | Lam.per | 5 | 0.625032 | 0.001654 |
| HT wk 1 | Width | 5 | 0.83994 | 0.001656 |
| HT wk 2 | Ep.thick | 7 | 1.034636 | 0.003816 |
| HT wk 2 | Lam.per | 6 | 0.640418 | 0.002078 |
| HT wk 2 | Width | 6 | 0.840613 | 0.002071 |
| HT wk 4 | Ep.thick | 7 | 1.023739 | 0.003816 |
| HT wk 4 | Lam.per | 4 | 0.615643 | 0.00198 |
| HT wk 4 | Width | 4 | 0.833079 | 0.001984 |
| HT wk 5 | Ep.thick | 6 | 1.032602 | 0.004122 |
| HT wk 5 | Lam.per | 5 | 0.617276 | 0.00149 |
| HT wk 5 | Width | 5 | 0.842557 | 0.001499 |

***Planned contrast analyses using FDR corrections (Pcutoff = 2.037e-6)***

|  | Treatment.1 | Measurement.1 | Treatment.2 | Measurement.2 | p.value | sig |
| --- | --- | --- | --- | --- | --- | --- |
| 1 | Control | Ep.thick | HT wk 0 | Ep.thick | 0.999477 | No |
| 2 | Control | Ep.thick | HT wk 1 | Ep.thick | 0.999431 | No |
| 3 | Control | Ep.thick | HT wk 2 | Ep.thick | 0.744403 | No |
| 4 | Control | Ep.thick | HT wk 4 | Ep.thick | 1 | No |
| 5 | Control | Ep.thick | HT wk 5 | Ep.thick | 0.963524 | No |
| 18 | HT wk 0 | Ep.thick | HT wk 1 | Ep.thick | 0.862461 | No |
| 19 | HT wk 0 | Ep.thick | HT wk 2 | Ep.thick | 0.250484 | No |
| 20 | HT wk 0 | Ep.thick | HT wk 4 | Ep.thick | 0.999847 | No |
| 21 | HT wk 0 | Ep.thick | HT wk 5 | Ep.thick | 0.544229 | No |
| 34 | HT wk 1 | Ep.thick | HT wk 2 | Ep.thick | 0.999999 | No |
| 35 | HT wk 1 | Ep.thick | HT wk 4 | Ep.thick | 0.999769 | No |
| 36 | HT wk 1 | Ep.thick | HT wk 5 | Ep.thick | 1 | No |
| 49 | HT wk 2 | Ep.thick | HT wk 4 | Ep.thick | 0.858378 | No |
| 50 | HT wk 2 | Ep.thick | HT wk 5 | Ep.thick | 1 | No |
| 63 | HT wk 4 | Ep.thick | HT wk 5 | Ep.thick | 0.983486 | No |
| 88 | Control | Lam.per | HT wk 0 | Lam.per | 0.862157 | No |
| 89 | Control | Lam.per | HT wk 1 | Lam.per | 1 | No |
| 90 | Control | Lam.per | HT wk 2 | Lam.per | 4.67E-07 | Yes |
| 91 | Control | Lam.per | HT wk 4 | Lam.per | 0.015769 | No |
| 92 | Control | Lam.per | HT wk 5 | Lam.per | 0.021596 | No |
| 99 | HT wk 0 | Lam.per | HT wk 1 | Lam.per | 0.921302 | No |
| 100 | HT wk 0 | Lam.per | HT wk 2 | Lam.per | 1.36E-10 | Yes |
| 101 | HT wk 0 | Lam.per | HT wk 4 | Lam.per | 0.920173 | No |
| 102 | HT wk 0 | Lam.per | HT wk 5 | Lam.per | 0.993725 | No |
| 109 | HT wk 1 | Lam.per | HT wk 2 | Lam.per | 2.04E-06 | Yes |
| 110 | HT wk 1 | Lam.per | HT wk 4 | Lam.per | 0.03314 | No |
| 111 | HT wk 1 | Lam.per | HT wk 5 | Lam.per | 0.054511 | No |
| 118 | HT wk 2 | Lam.per | HT wk 4 | Lam.per | 0 | Yes |
| 119 | HT wk 2 | Lam.per | HT wk 5 | Lam.per | 0 | Yes |
| 126 | HT wk 4 | Lam.per | HT wk 5 | Lam.per | 1 | No |
| 139 | Control | Width | HT wk 0 | Width | 1 | No |
| 140 | Control | Width | HT wk 1 | Width | 0.972593 | No |
| 141 | Control | Width | HT wk 2 | Width | 0.963549 | No |
| 142 | Control | Width | HT wk 4 | Width | 0.99802 | No |
| 143 | Control | Width | HT wk 5 | Width | 0.201172 | No |
| 144 | HT wk 0 | Width | HT wk 1 | Width | 0.998786 | No |
| 145 | HT wk 0 | Width | HT wk 2 | Width | 0.996716 | No |
| 146 | HT wk 0 | Width | HT wk 4 | Width | 0.992524 | No |
| 147 | HT wk 0 | Width | HT wk 5 | Width | 0.566262 | No |
| 148 | HT wk 1 | Width | HT wk 2 | Width | 1 | No |
| 149 | HT wk 1 | Width | HT wk 4 | Width | 0.416001 | No |
| 150 | HT wk 1 | Width | HT wk 5 | Width | 0.999489 | No |
| 151 | HT wk 2 | Width | HT wk 4 | Width | 0.436508 | No |
| 152 | HT wk 2 | Width | HT wk 5 | Width | 0.999999 | No |
| 153 | HT wk 4 | Width | HT wk 5 | Width | 0.018384 | No |

**R-routines:**

**Respirometry data (SMR, MMR, Aerobic Scope)**

**Libraries**

library(lme4)

library(multcomp)

library(lattice)

library(languageR)

library(LMERConvenienceFunctions)

my.control=lmerControl(optCtrl=list(maxfun=2000000))

**Models**

JJ1<-lmer(CoxboxO ~ Species*Treatment*Measurement + (Mass|Species:fish:Treatment), na.action=na.exclude, control=my.control, Jacob)

JJ2<-lmer(CoxboxO ~ 1 + (Mass|Species:fish:Treatment), na.action=na.exclude, control=my.control, Jacob)

anova(JJ1,JJ2)

JJ3<-lmer(CoxboxO ~ Species*Treatment*Measurement + (Mass|Species:fish), na.action=na.exclude, control=my.control, Jacob)

anova(JJ1,JJ3)

| JJ4<-lmer(CoxboxO ~ Species*Treatment*Measurement + (Mass\|fish), na.action=na.exclude,  control=my.control, Jacob)  anova(JJ3,JJ4) |
| --- |

**Assumptions**

shapiro.test(resid(JJ3))

qqnorm(residuals(JJ3))

bartlett.test(CoxboxO~Treatment,Jacob)

plot(fitted(JJ3),residuals(JJ3))

**Results**

pamer.fnc(JJ3)

lsmeans(JJ3, pairwise~Treatment*Measurement*Species, adjust=c("none"))

**FDR correction:**

sorted.pvalue<-sort(rawp1)

> sorted.pvalue

> j.alpha<-(1:45)*(.05/45)

> j.alpha

> diff<-sorted.pvalue-j.alpha

> diff

> neg.diff<-diff[diff<0]

> pos.diff<-neg.diff[length(neg.diff)]

> index<-diff==pos.diff

> index

> p.cutoff<-sorted.pvalue[index]

> p.cutoff

**Blood data:**

**Apogonid Blood Analyses**

**Models**

JJap<-lmer(Coxbox ~ Treatment*Tissue + (BM|Treatment:No), na.action=na.exclude, control=my.control, Jacob)

JJap1<-lmer(Coxbox ~ 1 + (BM|Treatment:No), na.action=na.exclude, control=my.control, Jacob)

anova(JJap,JJap1)

**Assumptions**

qqnorm(residuals(JJap))

bartlett.test(Coxbox~Treatment,Jacob)

**Results**

pamer.fnc(JJap)

lsmeans(JJap, pairwise~Treatment*Tissue, adjust=c("none"))

**FDR correction**

See code above

**Fusilera Blood Analyses**

**Models**

JJ6<-lmer(Coxbox ~ Treatment*Tissue + (BM|Treatment:No), na.action=na.exclude, control=my.control, Jacob)

JJ7<-lmer(Coxbox ~ 1 + (BM|Treatment:No), na.action=na.exclude, control=my.control, Jacob)

anova(JJ6,JJ7)

**Assumptions**

bartlett.test(Coxbox~Treatment,Jacob)

qqnorm(residuals(JJ6))

**Results**

pamer.fnc(JJ6)

lsmeans(JJ6, pairwise~Treatment*Tissue, adjust=c("none"))

**FDR correction**

See code above

**Gill data**

**Apogonid Gill Analyses**

**Models**

al1=lmer(Value~Trmt*Measurement+(1|Trmt:ID:Loci.gill:Filament:Loci.fil:Lamellae), na.action=na.omit, apo_min)

al2=lmer(Value~1+(1|Trmt:ID:Loci.gill:Filament:Loci.fil:Lamellae), na.action=na.omit, apo_min)

anova(al1,al2)

al3=lmer(Value~Trmt+Measurement+(1|Trmt:ID:Loci.gill:Filament:Loci.fil:Lamellae), na.action = na.omit, apo_min)

anova(al1,al3)

al4=lmer(Value~Trmt*Measurement+(1|Trmt:ID:Loci.gill:Filament:Loci.fil), na.action=na.omit, apo_min)

anova(al1,al4)

al5=lmer(Value~Trmt*Measurement+(1|Trmt:ID:Loci.gill:Filament), na.action=na.omit, apo_min)

anova(al4,al5)

anova(al4)

**Assumptions**

qqnorm(resid(al4))

plot(residuals(al4))

**Boxcox transformation**

bc=boxCox(lm(Value~Trmt*Measurement, data = apo_min), lambda = seq(-0.4, 0.1, 1/10), plotit = TRUE)

bc

lambda=bc$x

lik=bc$y

bc1=cbind(lambda, lik)

bc1

bc1[order(-lik),]

apo_min$boxcox=cbind(apo_min$Value^-0.091919192)

bc_apo=-0.091919192

**Boxcox models**

al1=lmer(boxcox~Trmt*Measurement+(1|Trmt:ID:Loci.gill:Filament:Loci.fil:Lamellae), na.action=na.omit, apo_min)

al2=lmer(boxcox~1+(1|Trmt:ID:Loci.gill:Filament:Loci.fil:Lamellae), na.action=na.omit, apo_min)

anova(al1,al2)

al3=lmer(boxcox~Trmt+Measurement+(1|Trmt:ID:Loci.gill:Filament:Loci.fil:Lamellae), na.action = na.omit, apo_min)

anova(al1,al3)

al4=lmer(boxcox~Trmt*Measurement+(1|Trmt:ID:Loci.gill:Filament:Loci.fil), na.action=na.omit, apo_min) ****This is the final model**

anova(al1,al4)

al5=lmer(boxcox~Trmt*Measurement+(1|Trmt:ID:Loci.gill:Filament), na.action=na.omit, apo_min)

anova(al4,al5)

**Assumptions**

qqnorm(resid(al4))

plot(residuals(al4))

**Results**

alsm4_min=lsmeans(al4, pairwise~Trmt*Measurement)

**FDR correction**

See code above

**Fusilera Gill Analyses**

**Models**

fl1=lmer(Value~Trmt*Measurement+(1|Trmt:ID:Loci.gill:Filament:Loci.fil:Lamellae), na.action=na.omit, rawf)

fl2=lmer(Value~1+(1|Trmt:ID:Loci.gill:Filament:Loci.fil:Lamellae), na.action=na.omit, rawf)

anova(fl1,fl2)

fl3=lmer(Value~Trmt+Measurement+(1|Trmt:ID:Loci.gill:Filament:Loci.fil:Lamellae), na.action = na.omit, rawf)

anova(fl1,fl3)

fl4=lmer(Value~Trmt*Measurement+(1|Trmt:ID:Loci.gill:Filament:Loci.fil), na.action=na.omit, rawf)

anova(fl1,fl4)

fl5=lmer(Value~Trmt*Measurement+(1|Trmt:ID:Loci.gill:Filament), na.action=na.omit, rawf)

anova(fl4,fl5)

anova(fl4)

**Assumptions**

qqnorm(resid(fl4))

plot(residuals(fl4))

**Boxcox transformation**

bc=boxCox(lm(Value~Trmt*Measurement, data = rawf), lambda = seq(-0.4, 0.1, 1/10), plotit = TRUE)

bc

lambda=bc$x

lik=bc$y

bc1=cbind(lambda, lik)

bc1

bc1[order(-lik),]

rawf$boxcox=cbind(rawf$Value^-0.152525253)

bc_fus=-0.152525253

**Boxcox transformed models**

fl1=lmer(boxcox~Trmt*Measurement+(1|Trmt:ID:Loci.gill:Filament:Loci.fil:Lamellae), na.action=na.omit, rawf)

fl2=lmer(boxcox~1+(1|Trmt:ID:Loci.gill:Filament:Loci.fil:Lamellae), na.action=na.omit, rawf)

anova(fl1,fl2)

fl3=lmer(boxcox~Trmt+Measurement+(1|Trmt:ID:Loci.gill:Filament:Loci.fil:Lamellae), na.action = na.omit, rawf)

anova(fl1,fl3)

fl4=lmer(boxcox~Trmt*Measurement+(1|Trmt:ID:Loci.gill:Filament:Loci.fil), na.action=na.omit, rawf)

anova(fl1,fl4) ****This is the final model**

fl5=lmer(boxcox~Trmt*Measurement+(1|Trmt:ID:Loci.gill:Filament), na.action=na.omit, rawf)

anova(fl4,fl5)

anova(fl4)

**Assumptions**

qqnorm(resid(fl4))

plot(residuals(fl4))

**Results**

flsm4_min=lsmeans(fl4, pairwise~Trmt*Measurement)

**FDR correction**

See code above
